# Dual-Site Acetylcholinesterase Inhibition and Multiscale Stability of Fused Quinoline Sulfonamides: A Chemoinformatic GA-MLR and Molecular Dynamics Study

**DOI:** 10.3390/ijms27073286

**Published:** 2026-04-04

**Authors:** Shrikant S. Nilewar, Apurva D. Chavan, Ankita R. Pradhan, Anshuman A. Tripathy, Nagaraju Bandaru, Prashik B. Dudhe, Perli Kranti Kumar, Sandesh Lodha, Ghazala Muteeb, Ivan Peredo-Valderrama, Antonio Jose Naranjo-Redondo, Tushar Janardan Pawar

**Affiliations:** 1 Department of Pharmaceutical Chemistry, Maliba Pharmacy College, Uka Tarsadia University, Bardoli 394350, Gujarat, India; shrinilewar@gmail.com (S.S.N.); sandeshlodha@gmail.com (S.L.); 2 School of Pharmaceutical Science, Sandip University, Nashik 422213, Maharashtra, India; apurvachavan237@gmail.com (A.D.C.); ankitap41103@gmail.com (A.R.P.); tripathyanshuman42@gmail.com (A.A.T.); dudhe.prashik@gmail.com (P.B.D.); 3 Department of Pharmacology, Sree Dattha Institute of Pharmacy Sheriguda, Ibrahimpatnam, Hyderabad 501510, Telangana, India; bnrajupharma@gmail.com; 4 Department of Pharmaceutical Analysis, J.K.K. Nattraja College of Pharmacy, Kumarapalayam 638183, Tamil Nadu, India; drpkk1987@gmail.com; 5 Department of Nursing, College of Applied Medical Sciences, King Faisal University, Al-Ahsa 31982, Saudi Arabia; graza@kfu.edu.sa; 6 Ingeniería en Sistemas Computacionales, Universidad Politécnica de Querétaro, Carretera Estatal 420 S/N, El Rosario, Santiago de Querétaro 76240, Querétaro, Mexico; ivan.peredo@upq.mx; 7 División de Ingeniería, Universidad Anáhuac Querétaro, Circuito Universidades I, Fracción 2 S/N, Zibatá, El Marqués 76246, Querétaro, Mexico; 8 Centro de Investigación, Universidad Anáhuac Querétaro, Circuito Universidades I, Fracción 2 S/N, Zibatá, El Marqués 76246, Querétaro, Mexico

**Keywords:** acetylcholinesterase, QSAR, molecular dynamics, fused quinolines, drug discovery, Alzheimer’s disease, global health

## Abstract

Alzheimer’s disease (AD) represents an escalating global neuropharmacological crisis, with prevalence in high-growth demographic regions such as India projected to exceed 14 million by 2040. This study addresses the urgent need for high-potency, dual-site acetylcholinesterase (AChE) inhibitors through an integrated computational pipeline. We address the failure of mono-target paradigms by designing scaffolds capable of simultaneously anchoring the Catalytic Active Site (CAS) and the Peripheral Anionic Site (PAS). A robust GA-MLR QSAR model was developed from 115 quinoline analogs using 11,135 descriptors. Lead candidates were prioritized via cavity directed molecular docking (7XN1) and 100 ns molecular dynamics (MD) simulations. The five-descriptor model (*R*^2^ = 0.7569, $Q_{LOO}^{2}$ = 0.7244) was validated by an external set of 8 experimental compounds ($R_{ext}^{2}$ = 0.8620). Lead Compound **19** emerged as a superior candidate (Δ*G* = −11.1 kcal/mol), exhibiting a stable MD trajectory (PL-RMSD ≈ 2.4 Å) and preserving essential Gly121-His447 catalytic anti-correlations. This study provides a statistically validated scaffold and computational mechanistic foundation for future in vitro experimental validation, advancing the high throughput screening of neuroprotective agents on a global scale.

## 1. Introduction

**Figure 1:**
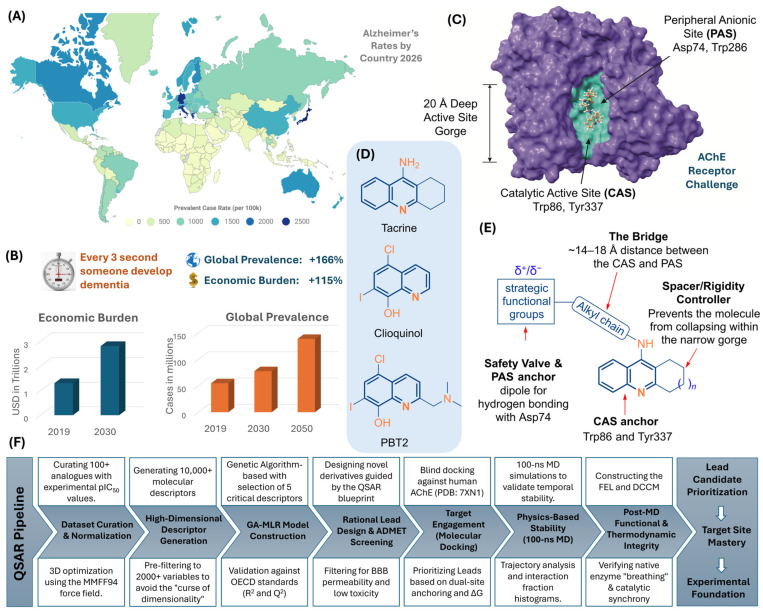
Strategic Rationale, Structural Challenges, and Chemoinformatic Discovery Pipeline. (**A**) Global Alzheimer’s Prevalence (2026): Heatmap of country-level dementia rates; source: © World Population Review (2026), accessed 3 February 2026 under CC BY license [3]. (**B**) Escalating Dementia Burden: Current and projected global statistics [4]. (**C**) The 20-Å Active-Site Challenge: Topographical anatomy of human AChE (PDB 7XN1) depicting the physical distance between the Catalytic Active Site (CAS) and the Peripheral Anionic Site (PAS). (**D**) Current market leads: Tacrine, Clioquinol and PBT2. (**E**) Scaffold Advantage: Strategic design of the fused quinoline core. (**F**) Integrated QSAR Logic of Discovery pipeline.

The global demographic transition toward an increasingly elderly population has precipitated an unprecedented neuropharmacological crisis, with Alzheimer’s disease (AD) emerging as the most significant driver of dementia worldwide. Currently accounting for approximately 60–70% of the 55 million global cases of dementia, AD is projected to affect nearly 139 million individuals by 2050 (Figure 1A,B) [1,2,3,4]. This escalation is not merely a clinical statistic; it represents a looming economic catastrophe. The global cost of dementia care was valued at USD 1.3 trillion in 2020 and is anticipated to double to USD 2.8 trillion by 2030, potentially destabilizing health systems across both high-income and developing nations (Figure 1B) [3,4]. Despite decades of intense investigation, the therapeutic landscape remains dominated by symptomatic treatments that fail to alter the underlying neurodegenerative trajectory, leaving a profound void in disease-modifying interventions [5].

The recent clinical disillusionment following the controversial approval and subsequent efficacy concerns of amyloid-targeted monoclonal antibodies, such as aducanumab and lecanemab, has highlighted the inherent limitations of mono-target paradigms [6]. These high-cost biological therapies have been plagued by significant safety issues, specifically Amyloid-Related Imaging Abnormalities (ARIA), characterized by brain edema (ARIA-E) and micro-hemorrhages (ARIA-H) [7]. Beyond primary neurodegenerative triggers, the pharmacological landscape is further complicated by recent pharmacovigilance data from the FDA Adverse Event Reporting System, which established concerning associations between common medications, such as proton pump inhibitors, and increased dementia events. This multifactorial complexity underscores the urgent necessity for novel therapeutics that possess superior safety profiles and high-occupancy target engagement to mitigate the cumulative burden on the aging brain [8]. Consequently, this shift in perspective has reinvigorated scientific interest in the cholinergic hypothesis, specifically repositioning the enzyme acetylcholinesterase (AChE) as a multifunctional structural target. While AChE typically facilitates the rapid hydrolysis of the neurotransmitter acetylcholine (ACh) within the synaptic cleft, the AD brain suffers from a profound ACh deficit that is catastrophically compounded by sustained enzymatic activity [9,10].

The structural anatomy of the human AChE enzyme provides a sophisticated, albeit challenging, blueprint for drug design (Figure 1C). The enzyme contains a narrow, 20-Å-deep active-site gorge with two distinct binding domains: the Catalytic Active Site (CAS) at the base and the Peripheral Anionic Site (PAS) at the entrance [11,12]. Traditional inhibitors, such as the first-generation agent Tacrine, primarily target the CAS to provide transient symptomatic relief by preserving available acetylcholine pools (Figure 1D) [13]. However, the therapeutic landscape has evolved toward identifying multi-target scaffolds, as seen with experimental leads like Clioquinol and PBT2, which aim to address metal-mediated neurotoxicity alongside enzymatic inhibition (Figure 1D) [14]. Recent biochemical evidence has established that the PAS serves as more than a simple gatekeeper; it acts as a pro-aggregating modulator of β-amyloid (Aβ) peptide aggregation [15,16]. The interaction between the PAS and Aβ peptides accelerates the formation of neurotoxic fibrils, creating a pathological bridge between cholinergic signaling failure and the amyloidogenic cascade [16,17]. This dual functionality makes AChE a pivotal target for “dual-site” inhibitors, molecules designed to simultaneously anchor at the CAS to preserve ACh and blockade the PAS to halt amyloid aggregation [18,19].

Despite the conceptual elegance of dual-site inhibition, a critical research gap persists in the rational design of novel ligands that possess optimized Absorption, Distribution, Metabolism, Excretion, and Toxicity (ADMET) profiles [20]. Existing drugs like Tacrine were withdrawn from clinical use primarily due to severe, dose-dependent hepatotoxicity, a liability that remains a significant hurdle for fused quinoline scaffolds [21]. Furthermore, the Blood–Brain Barrier (BBB) represents a formidable pharmacological obstacle; many ligands that exhibit potent in vitro activity fail to achieve therapeutically relevant concentrations in the Central Nervous System (CNS) [20,21]. This necessitates a transition toward specialized neuroprotective scaffolds, such as the generalized fused quinoline scaffold (Figure 1E), which allows for the exploration of diverse functional groups at the PAS-anchoring site.

The convergence of chemoinformatics and biomimetic chromatography represents a powerful new trend in drug discovery, offering high-throughput alternatives to unethical animal testing [22,23]. Biomimetic chromatography, utilizing stationary phases like Immobilized Artificial Membranes (IAM) and Human Serum Albumin (HSA), provides experimentally grounded predictors of drug disposition by mimicking the polar-apolar interfaces of cell membranes and protein-binding environments [24]. By integrating these experimental parameters with sophisticated Quantitative Structure-Activity Relationship (QSAR) modeling, researchers can establish reliable correlations between molecular topology and biological fate [25]. In this context, the use of advanced descriptors, such as constitutional indices for conjugation assessment and GETAWAY descriptors for longitudinal reach enables a level of structural mapping previously unattainable. However, static QSAR models often fail to account for the induced-fit motions and conformational rearrangements inherent in protein-ligand interactions, necessitating the inclusion of physics-based molecular dynamics (MD) to validate the temporal stability of the predicted binding modes [26].

The development of specialized neuroprotective scaffolds that align with these standards is essential for fostering a self-sustaining scientific ecosystem. Current literature reveals a lack of comprehensive pipelines that combine large-scale descriptor pools, GA-MLR-based feature selection and long-range dynamic simulations to optimize fused ring systems for dual-site AChE engagement [27,28,29]. Bridging this gap is critical for the discovery of next-generation, disease-modifying AD therapeutics that are both clinically effective and toxicologically safe.

In this work, we address these multifaceted challenges through a rigorous computational optimization pipeline focused on novel fused quinoline sulfonamides (Figure 1F). By curating a diverse dataset of 115 analogs and generating 11,135 molecular descriptors, we aim to develop a robust GA-MLR QSAR model that satisfies the stringent OECD standards for statistical validity and interpretability. The focus is directed toward identifying the primary structural drivers, such as π-conjugation length and spatial mass distribution that allow a ligand to effectively bridge the distance between the CAS and PAS within the human AChE gorge. We prioritize the design of 16 novel derivatives guided by the resulting QSAR equation, subjecting the most promising leads to ADMET screening and cavity directed molecular docking against the human AChE crystal structure (7XN1). The ultimate goal is to evaluate the dynamic stability and structural integrity of these lead-enzyme complexes through 100 ns molecular dynamics simulations and Dynamic Cross-Correlation Matrix (DCCM) analysis. By integrating quantitative modeling with physics-based dynamic analysis, this study provides a validated framework for the discovery of anti-Alzheimer’s agents that satisfy the requirements for scientific sovereignty and frontline neurodegenerative research.

## 2. Results and Discussion

### 2.1. QSAR Model Development and Statistical Robustness

A quantitative structure-activity relationship (QSAR) model was developed to predict the acetylcholinesterase (AChE) inhibitory activity of fused-ring quinoline analogs using multiple linear regression (MLR). Genetic algorithm-based feature selection was employed to prioritize five molecular descriptors from an initial pool of 11,135 variables, identifying *max_conj_path*, *MATS3s*, *R8m*, *C-N-C=O*, and the *MNA* substructure *-H(-C(C-H-H-C))* as critical drivers of potency (Table S2). The resulting GA-MLR model, constructed from a training set of 81 compounds (Table S3), is defined by the following equation:

$${pIC}_{50}=4.9509+0.0554\cdot max\text{\_}conj\text{\_}path+5.3355\cdot MAT53s+2.0399\cdot R8m+1.186\cdot (C\text{-}N\text{-}C\text{=}O)+1.5020\cdot MNA$$

**Figure 2:**
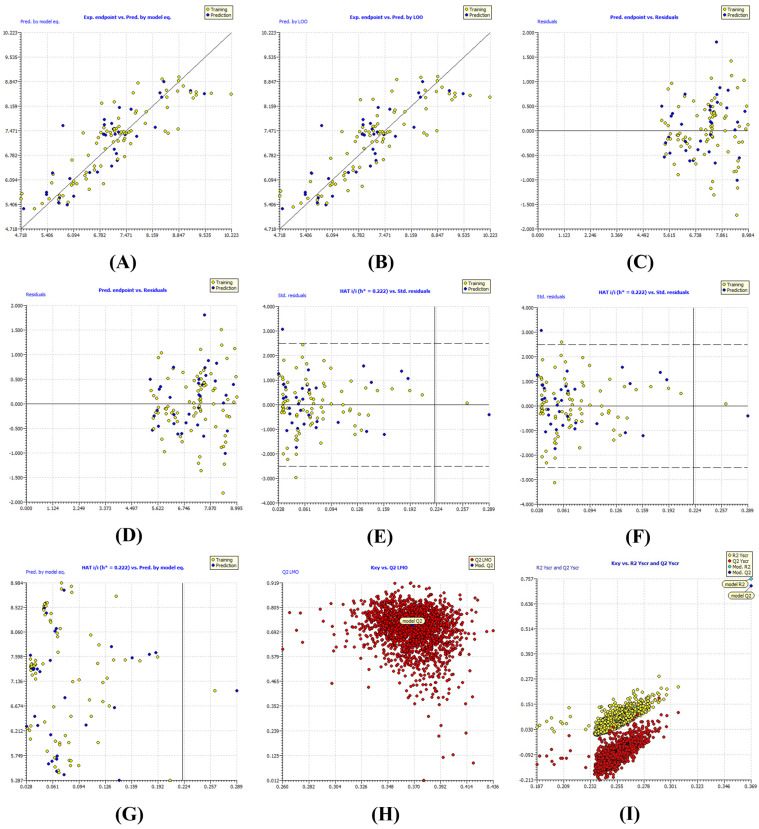
QSAR model validation diagnostics: (**A**,**B**) Experimental vs. Predicted activity plots for the Training Set (*n* = x) and External Test Set (*n* = y); (**C**,**D**) residual distribution analysis showcasing error homoscedasticity; (**E**,**F**) cross-validation Williams plots (LOO and LMO) for internal robustness; (**G**) Overall Applicability Domain (Williams plot) defining the warning leverage (δ*) and outlier detection limits; (**H**) LMO analysis (Q^2^ vs. R^2^ stability); (**I**) Y-randomization clustering confirming the absence of chance correlations.

The model demonstrated robust statistical validity, explaining approximately 76% of the variance in the training set (*R*^2^ = 0.7569; $R_{adj}^{2}$ = 0.7407). The predictive accuracy was supported by acceptable error metrics (RMSE = 0.5752; MAE = 0.4367) and an adequate concordance correlation coefficient (CCC = 0.8616). Internal validation using leave-one-out (LOO) and leave-many-out (LMO) cross-validation yielded *Q*^2^ values of 0.7244 and 0.7178, respectively. The negligible difference between *R*^2^ and $Q_{LOO}^{2}$ (Δ*R*^2^ = 0.0325) indicates high model stability and the absence of significant overfitting (Table 1). The model’s robustness was extensively evaluated through the visual diagnostics presented in Figure 2. The Experimental vs. Predicted activity plots (Figure 2A,B) demonstrate a linear distribution along the ideal diagonal, confirming reliable predictive fidelity across the potency range. To further scrutinize the error distribution, residual analysis was performed (Figure 2C,D). The random dispersion of residuals around the zero-horizontal axis indicates an unbiased model, proving that the errors are independent of the predicted potency values and confirming the suitability of the linear regression approach.

**Table 1:** Statistical validation parameters for the generated GA-MLR QSAR model.

Metric	QSAR Model	Threshold
*R*^2^ (training)	0.7569	>0.60
Adjusted *R*^2^	0.7407	Close to *R*^2^
RMSE (training)	0.5752	Lower is better
MAE (training)	0.4367	Lower is better
*Q*^2^ (LOO)	0.7244	>0.50
*Q*^2^ (LMO)	0.7178	>0.60
CCC (training)	0.8616	>0.85
$R_{pred}^{2}$ (predictive, *n* = 34)	0.737	>0.60
CCC (external)	0.8509	>0.85
$r_{m}^{2}$ (average)	0.6257	>0.50
$\Delta r_{m}^{2}$	0.0996	<0.20

*R*^2^: coefficient of determination (*n* = 81 training set); RMSE: root mean square error; MAE: mean absolute error; *Q*^2^: cross-validated *R*^2^ (LOO: leave-one-out; LMO: leave-many-out); CCC: concordance correlation coefficient; Δ$r_{m}^{2}$: proximity metric for observed vs. predicted values; $R_{pred}^{2}$: internal predictive validation of the 115-compound pool.

The Applicability Domain (AD) was delineated via a Williams plot (Figure 2G) to verify the model’s reliability for prospective lead design. The leverage analysis identified a threshold of h* = 0.222. Over 98% of the compounds remained within this threshold, with standardized residuals consistently localized within the ±3σ range. Two compounds exhibited leverage values exceeding h*, yet they maintained low residuals and were thus categorized as influential points that extend the model’s chemical space rather than outliers. This confirms that the model is well-equipped to predict the potency of the novel quinoline sulfonamides described later in this study.

Finally, the possibility of chance correlation was ruled out via 2000 iterations of Y-randomization (Figure 2I). The sharp decline in statistical performance ($R_{scr}^{2}$ = 0.0622) and the resulting negative cross-validation value ($Q_{scr}^{2}$ = −0.0961) serve as definitive proof that the model is structurally anchored. The LMO stability plot (Figure 2H) further confirms that the model’s performance is not dependent on a specific subset of data but is a consistent representation of the structure-activity relationship across the entire fused quinoline family.

### 2.2. Mechanistic Interpretation of Molecular Descriptors

The interpretability of the GA-MLR model is derived from the structural relevance of its five constituent descriptors, which characterize the macroscopic electronic, topological, and fragmental properties correlated with AChE inhibition. While MLR provides a purely statistical relationship, these descriptors allow us to formulate hypotheses regarding the physical requirements for binding within the human AChE active-site gorge, which are subsequently explored via molecular docking.

- ***max_conj_path* (alvaDesc):** This constitutional descriptor quantifies the length of the longest continuous π-conjugation path within the molecule. The positive coefficient identifies extended π-systems as primary potency drivers. From a structural perspective, this suggests a requirement for extensive aromatic surface area, which we hypothesize favors non-covalent π-π stacking interactions with the aromatic residues lining the enzyme gorge.
- ***MATS3s* (Dragon7):** This 2D-autocorrelation index represents Moran autocorrelation of lag 3 weighted by the intrinsic state (I-state). It describes the electronic distribution and polarization across the molecular graph at a specific topological distance. In the context of AChE, appropriately distributed electronic density is statistically linked to higher potency, likely by providing the necessary electrostatic profile for favorable interactions within the gorge.
- ***R8m* (Dragon7):** As a GETAWAY (Geometry, Topology, and Atom Weights AssemblY) descriptor, *R8m* denotes the *R* autocorrelation of lag 8 weighted by mass. This topological index captures the longitudinal reach and mass distribution of the scaffold. Its selection as a major potency driver statistically supports the dual-site inhibition paradigm, suggesting that molecules must possess a specific longitudinal extension to effectively span the macroscopic distance between the catalytic and peripheral binding sites.
- ***C-N-C=O* (Fragmentor):** This fragment-based descriptor identifies the presence of carbamoyl or urea-like functional groups. Moieties identified by this descriptor act as essential hydrogen-bond donors or acceptors. The model indicates these polar features are statistically significant for potency, providing necessary pharmacophoric points for polar contacts within the active site.
- ***MNA* (Multilevel Neighborhoods of Atoms):** Multilevel Neighborhoods of Atoms (*MNA*) are 2D-substructural notations that describe the local environment of an atom. This specific *MNA* string accounts for subtle steric and hydrophobic effects in methyl-bearing local environments. Its inclusion suggests that potency is statistically sensitive to substitution patterns that modulate the local atomic neighborhood, likely influencing the van der Waals fit within the hydrophobic mid-gorge.

These five descriptors demonstrate that inhibitory potency is not a simple function of lipophilicity but is precisely governed by the spatial arrangement of aromatic density, electronic distribution, and fragmental reach. This mechanistic foundation provides the rational basis for the structural modifications used to design the high-potency leads described in the subsequent sections.

### 2.3. External Validation and Structural Generalizability

The definitive metric for the utility of a QSAR model in prospective drug design is its predictive fidelity when applied to unseen chemical entities. To confirm the generalizability of the five-descriptor GA-MLR model, an external validation set of eight compounds (*n* = 8) was utilized (Table 2). These compounds, which were excluded from the initial training and feature selection phases, provide a rigorous structural “checkpoint” to verify that the model captures genuine biological interactions rather than local statistical artifacts [30,31,32,33].

While the strictly independent external validation set (*n* = 8) is numerically concise, it was not utilized as the sole metric of model generalizability (Table 2); the primary predictive robustness was established by the 34-compound internal prediction set. Rather, this 8-compound external set was purposefully curated as a secondary, highly demanding statistical ‘stress test’. These compounds encompass structurally distinct chemical domains (e.g., substituted tetrahydronaphthyridines and pyrazolo-pyrano-pyridines) and span a massive 6-log biological potency range, from low-micromolar (pI_50_ 4.8) to sub-nanomolar (pI_50_ 10.7). Achieving a high correlation (R^2^_ext_ = 0.8620) across such extreme chemical and potency variance demonstrates that the model captures universal binding physics rather than interpolating local statistical artifacts.

The external validation yielded a coefficient of determination ($R_{ext}^{2}$) of 0.8620, indicating that the model successfully accounts for approximately 86% of the variance in the biological activity of the independent set. As illustrated in the regression profile (Figure 2), the predicted pIC_50_ values demonstrate a strong linear correlation with experimental activities. The distribution of these compounds is particularly significant; they span a wide activity spectrum from low-micromolar (pIC_50_ ≈ 4.8) to sub-nanomolar (pIC_50_ ≈ 10.7) potencies, indicating that the model maintains predictive consistency even at high-potency extremes where the 20 Å AChE gorge becomes increasingly sensitive to structural nuances.

**Table 2:** Experimental versus calculated pIC_50_ values for the external validation set (*n* = 8).

Compound	1	2	3	4	5	6	7	8
Calculated pIC_50_	4.782	5.288	8.429	7.620	6.272	6.024	6.306	7.314
Experimental pIC_50_	4.849	5.070	10.700	7.469	6.066	6.143	6.857	7.221
Reference	[30]	[31]	[31]	[30]	[30]	[32]	[32]	[33]

pIC_50_ = −log_10_(IC_50_ [M]). Experimental activity values obtained from standardized literature datasets.

**Figure 3:**
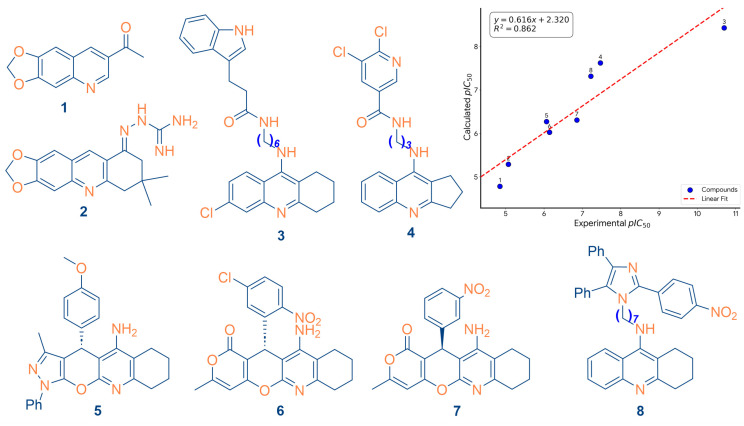
External validation profile of the GA-MLR model: (left) structural architecture of the eight validation Compounds (**1**–**8**) representing the chemical diversity used to test the model; (right) regression analysis showing the high correlation between Experimental and Calculated pIC_50_ (*R*^2^ = 0.862) [30,31,32,33].

The structural architecture of the validation set (Figure 3, left panel) encompasses diverse fused-ring scaffolds, including substituted tetrahydronaphthyridines, pyrazolo-pyrano-pyridines, and indole-quinoline hybrids. The successful prediction of activity across these chemically distinct frameworks validates the robustness of the selected descriptors, specifically *max_conj_path* and *R8m*. This confirms that the model’s identification of extended π-conjugation and longitudinal reach as potency drivers is a universal requirement for human AChE inhibition within the quinoline chemical family. While the regression slope (*y* = 0.616*x* + 2.320) indicates a slight compression of the predicted range, the absence of pronounced systematic deviations or outliers confirms the model’s suitability for the prospective lead prioritization discussed in the following section.

### 2.4. Design and Predictive Profiling of Novel Fused Quinolines

The successful validation of the GA-MLR model established a quantitative blueprint for the rational optimization of the fused quinoline scaffold. In this phase, we applied the model equation to a prospective library of eighteen analogs (*n* = 18) designed to explore the structural requirements for enhanced dual-site inhibition. To ensure predictive calibration and maintain scientific integrity, the library was structured to include sixteen novel derivatives and two internal benchmarks (Compounds **2** and **4**) whose experimental activities were utilized in the previous validation phase.

The design strategy focused on the synergistic modulation of the three primary potency drivers identified by the model: π-conjugation length (*max_conj_path*), electronic density distribution (*MATS3s*), and longitudinal mass distribution (*R8m*). Structural modifications primarily involved the introduction of sulfonamide and extended amine spacers to bridge the 20-Å enzyme gorge while maintaining favorable drug-like attributes. The predicted pIC_50_ values for the designed series, summarized in Table 3, fall within a chemically meaningful range relative to the training set, with several novel leads exhibiting activities comparable to the high-potency benchmarks.

**Table 3:** Rational design, descriptor-guided modifications, and predicted inhibitory activities (pIC_50_) for the optimized fused quinoline series.

Compd.	Modifications Done	Descriptors Used	Rationale Behind Structure Design	pIC_50_
**2**	Fused quinoline with alkyl substitutions and ester groups	*max_conj_path*, *MATS3s*, *C-N-C=O*	Alkyl and ester groups introduce flexibility and increase the complexity of molecular geometry, impacting polarity and size.	4.3725
**4**	Fused quinoline with amine, halogen, and ester groups	*max_conj_path*, *MATS3s*, *R8m*	Amine, halogen, and ester substitutions influence molecular shape, electronic properties, and size.	8.0588
**9**	Fused quinoline with alkyl and halogen substitutions	*max_conj_path*, *MATS3s*, *R8m*	Fused quinoline rings enhance conjugation, while alkyl and halogen substitutions influence lipophilicity and molecular shape.	6.1265
**10**	Fused quinoline attached to a benzene ring with ester groups	*max_conj_path*, *MATS3s*, *R8m*, *C-N-C=O*	Ester and benzene ring groups increase topological complexity, modifying polarity and molecular geometry.	5.9286
**11**	Fused quinoline with ester and amine substitutions	*max_conj_path*, *MATS3s*, *R8m*	Ester and amine substitutions modify molecular shape and increase flexibility, altering lipophilicity and geometry.	6.3097
**12**	Fused quinoline with extended amines and halogen substitutions	*max_conj_path*, *MATS3s*, *C-N-C=O*	Extended amines and halogen groups introduce greater flexibility and polarity, affecting molecular interactions.	7.4803
**13**	Fused quinoline with extended carbon chains and halogen	*max_conj_path*, *MATS3s*, *R8m*	Longer alkyl chains and halogen substitutions alter hydrophobicity and increase molecular complexity.	7.5403
**14**	Extended quinoline with additional carbon chains and halogen	*max_conj_path*, *MATS3s*, *C-N-C=O*	Additional carbon chains and halogen groups increase hydrophobicity and modify molecular geometry for better structural balance.	7.5616
**15**	Extended quinoline fused with a cyclic group, with ester substitutions	*max_conj_path*, *MATS3s*, *R8m*, *MNA*	Cyclic structures and ester substitutions modify the molecular geometry, improving structural complexity and hydrophobicity.	5.6982
**16**	Extended quinoline with multiple substitution groups	*max_conj_path*, *MATS3s*, *R8m*, *MNA*	Multiple substitutions alter the topological structure, increasing rigidity and adjusting molecular shape.	5.3384
**17**	Fused quinoline with additional nitrogen substitutions	*max_conj_path*, *MATS3s*, *R8m*, *C-N-C=O*	Nitrogen substitutions influence polarity and the complexity of the molecular structure, modifying its topology.	5.3398
**18**	Fused quinoline with nitrogen-containing functionalization	*max_conj_path*, *MATS3s*, *R8m*, *MNA*	Nitrogen-containing functional groups increase polarity and modify the shape of the molecule for better structural interaction.	5.1299
**19**	Fused quinoline with sulfonate group and amines	*max_conj_path*, *MATS3s*, *R8m*, *MNA*	Sulfonate groups add polarity, while amines introduce additional flexibility and complexity to the molecular structure.	5.8935
**20**	Sulfonate substituted quinoline fused with amine groups	*max_conj_path*, *MATS3s*, *R8m*, *MNA*	Sulfonate and amine substitutions affect polarity and increase the complexity of the molecular structure.	5.9529
**21**	Additional functional groups and fused rings	*max_conj_path*, *MATS3s*, *C-N-C=O*	Fused rings and additional functional groups introduce rigidity, modifying the shape and flexibility of the molecule.	7.1775
**22**	Fused quinoline with extended ring systems and ester functionalities	*max_conj_path*, *MATS3s*, *R8m*	Extended ring systems and ester groups provide additional rigidity and alter molecular flexibility.	7.7726
**23**	Extended quinoline with ester and oxygen-containing functional groups	*max_conj_path*, *MATS3s*, *R8m*, *C-N-C=O*	Ester and oxygen-containing groups enhance polarity and hydrophobicity, modifying the molecular geometry for better flexibility.	7.1909
**24**	Fused quinoline with halogen, ester, and amine groups	*max_conj_path*, *MATS3s*, *R8m*, *MNA*	Halogen, ester, and amine groups increase hydrophobicity and polarity, modifying the overall shape and topological complexity.	7.0912

*max_conj_path*: maximum length of π-conjugation path; *MATS3s*: Moran autocorrelation of lag 3 weighted by I-state; *R8m*: GETAWAY R-autocorrelation of lag 8 weighted by mass; *MNA*: Multilevel Neighborhood of Atoms substructure *-H(-C(C-H-H-C))*.

**Figure 4:**
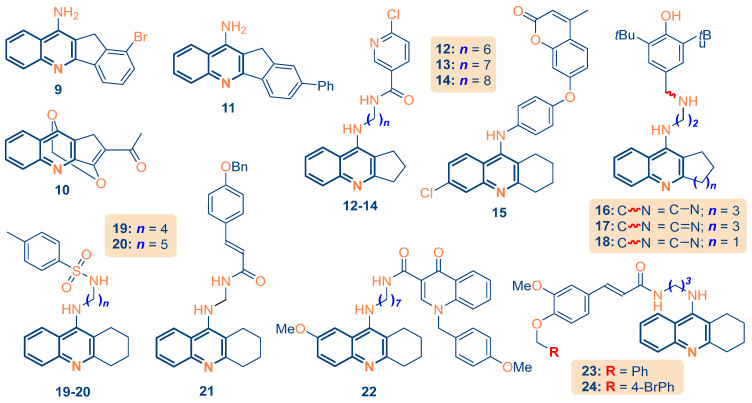
Structural architecture and chemical space distribution of the 16 novel fused quinoline analogs with 2 internal benchmarks (**2** and **4**) used for model calibration.

A critical observation from this screening was that while several novel leads exhibited predicted potencies similar to the benchmarks, Lead Compounds **19** and **20** were prioritized for subsequent physics-based evaluation. The rationale for this selection was not raw activity alone, but the achievement of superior drug-like attributes. The sulfonamide moiety in **19** and **20** provides a critical dipole for PAS interaction while maintaining synthetic accessibility (*Synth* ≈ 2.2) and a safety profile that distinguishes them from the literature benchmarks. While several novel leads (e.g., Compounds **12**, **14**, and **22**) exhibited higher predicted potencies, Leads **19** and **20** were prioritized using a strict Multi-Parameter Optimization (MPO) framework to balance viable baseline potency with superior synthetic accessibility (Synth ≈ 2.2), optimal BBB permeability, and significantly reduced off-target toxicity profiles. As shown in Figure 4, the entire design series resides within the model’s applicability domain, ensuring that the prospective predictions are statistically sound.

### 2.5. ADMET Screening and Toxicity Observations

The prioritization of Lead Compounds **19** and **20** from the designed series was predicated on a multi-parameter evaluation of their pharmacokinetic (ADME) profiles and toxicological (T) risks. In neurodegenerative drug discovery, high inhibitory potency is therapeutically irrelevant if the scaffold lacks the physicochemical attributes necessary to traverse the Blood–Brain Barrier (BBB) or exhibit prohibitive systemic toxicity. The comparative ADMET parameters for Leads **19**, **20** (Figure S1), and the clinical reference drug Tacrine are summarized in Table 4.

**Table 4:** Comparative ADMET parameters and predicted docking affinities for prioritized Leads (**19** and **20**) versus Tacrine.

Parameter	19	20	Tacrine
QED	0.520	0.463	0.706
logS (log mol/L)	−4.698	−4.898	−2.875
*Synth*	2.268	2.292	2.015
caco2 (log cm/s)	−4.956	−5.000	−4.677
logD	3.703	3.748	2.101
BBB	0.647	0.768	0.977
logP	4.385	4.556	2.432
ΔG (kcal/mol)	−11.1	−10.6	−9.0

QED: Quantitative Estimate of Drug-likeness; logS: aqueous solubility (mol/L); *Synth*: Synthetic Accessibility Score (1 = easy, 10 = difficult); caco2: caco-2 cell line permeability (cm/s); logD: distribution coefficient at pH 7.4; BBB: Blood–Brain Barrier score (>0.3 suggests CNS activity); logP: cctanol-water partition coefficient; ΔG: Gibbs free energy of binding against PDB 7XN1.

Prioritized Lead **19** and **20** demonstrate exceptional Blood–Brain Barrier (BBB) permeability scores (0.647 and 0.768, respectively), which successfully meet the threshold for CNS-targeted therapeutics. While the reference drug Tacrine exhibits a higher BBB score of 0.977 and superior aqueous solubility (logS −2.875), it possesses a significantly lower lipophilicity (logP 2.432) compared to the novel leads (logP 4.38–4.55). The increased lipophilic character of the novel leads is a strategic requirement to optimize π-stacking interactions within the hydrophobic 20-Å AChE gorge. Furthermore, the Synthetic Accessibility (*Synth*) scores of ≈2.2 for the novel leads are comparable to Tacrine (2.015), indicating that these scaffolds are easily accessible for large-scale production despite their increased structural complexity. Notably, Lead 19 exhibited a superior QED score (0.520) compared to 20 (0.463), suggesting a more balanced drug-like architecture for the *n*-butyl sulfonamide linker.

Notably, Lead **19** exhibited a superior QED score (0.520) compared to 20 (0.463), suggesting a more balanced drug-like architecture. However, it is important to note that the predicted aqueous solubility (log S ≈ −4.7) and Caco-2 permeability (≈−5.0) for both leads are categorized as moderate. While these values represent borderline pharmacokinetic hurdles typical of large, lipophilic dual-site inhibitors, they remain within the manageable range for CNS-targeted lead optimization. These in silico metrics serve as early-stage comparative guides, and we acknowledge that subsequent formulation strategies, such as salt formation or nano-emulsification will be required during in vitro validation to address these specific solubility limitations.

Toxicological profiling using ProTox analysis revealed that while the quinoline core maintains a CYP2E1-mediated hepatotoxicity risk similar to Tacrine, the novel leads offer an improved safety window. Compounds **19** and **20** exhibited lower predicted probabilities for neurotoxicity and cardiotoxicity compared to the reference drug. By acknowledging the trade-off between increased lipophilicity for π-stacking and the resulting moderate solubility, we present these sulfonamide-linked quinolines as ‘safe-by-design’ candidates. This balanced approach prioritizes reduced systemic liabilities over maximal solubility, setting a realistic framework for the development of next-generation AChE inhibitors.

### 2.6. Molecular Docking and Active-Site Gorge Interaction

Molecular docking against the human AChE crystal structure (7XN1) validated the dual-site binding hypothesis for the prioritized sulfonamides. Both prioritized Leads **19** (Δ*G* = −11.1 kcal/mol) and **20** (Δ*G* = −10.6 kcal/mol) achieved significantly higher binding affinities than the clinical reference drug Tacrine (Δ*G* = −9.0 kcal/mol). To delineate the structural basis for this superiority, the binding mode of Tacrine was analyzed as a baseline reference (Tables S5 and S6). As illustrated in the interaction fingerprint, Tacrine functions strictly as a single-site inhibitor, anchoring the Catalytic Active Site (CAS) via π-π stacking with Trp86 and a conventional hydrogen bond with Ser125. Crucially, Tacrine lacks the molecular length required to engage the Peripheral Anionic Site (PAS), leaving the Asp74 residue unoccupied. To bridge the statistical predictions of the QSAR model with atomistic structural insights, we utilized molecular docking to evaluate the relative binding orientations of the identified leads. While docking scores (ΔG) provide a theoretical enthalpy-based ranking, they are employed here to corroborate the structural requirements identified by the topological descriptors, rather than as absolute thermodynamic correlates to the empirical pIC_50_ values.

**Figure 5:**
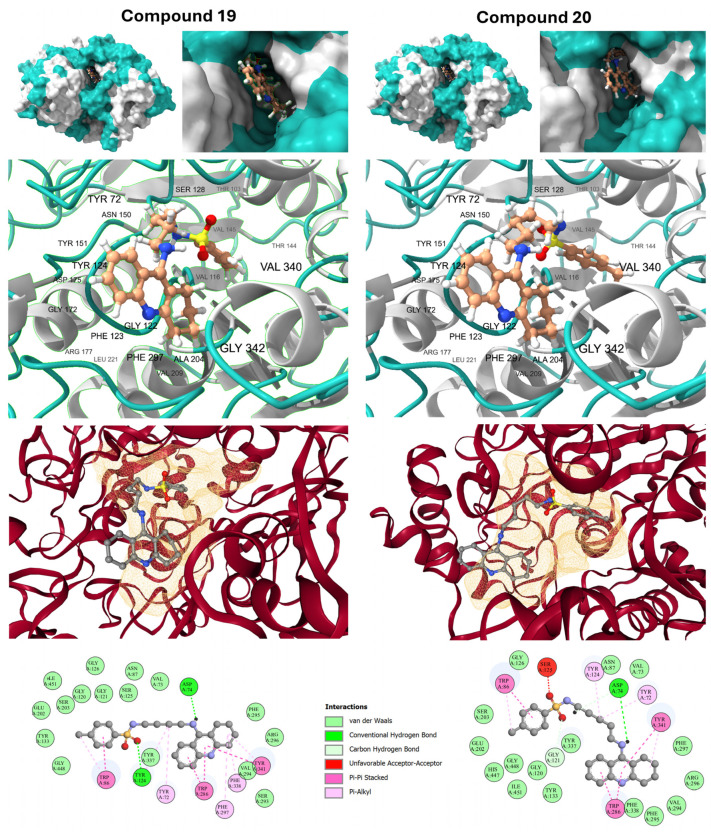
Comparative molecular docking analysis of Lead Compounds **19** and **20** within the human AChE active site (PDB ID: 7XN1). (Row 1) Surface representations and active-site gorge occupancy; (Row 2) Zoomed-in 3D orientations highlighting critical residue contacts (Tyr72, Tyr124, Phe297, Val340, etc.); (Row 3) Ribbon models showing ligand placement within the narrow enzyme gorge; (Row 4) 2D interaction maps delineating H-bonds (green), π-π stacking (magenta), and the unfavorable contact (red bubble) in Compound **20**.

Lead **19** demonstrated an optimized interaction fingerprint that effectively bridges the 20 Å enzyme gorge. As illustrated in the 2D interaction map (Figure 5), the tricyclic aromatic core anchors the Catalytic Active Site (CAS) via robust π-π stacking interactions with Trp86 and Tyr337, the primary residues responsible for acetylcholine recognition. Unlike Tacrine, the sulfonamide-linked *n*-butyl tail of Lead **19** extends toward the PAS, where it forms a critical, high-occupancy hydrogen bond with Asp74. This dual anchoring is further stabilized by π-π interactions with the PAS gatekeepers Trp286 and Tyr341, along with hydrophobic π-alkyl contacts with Phe338 and Tyr124. This binding mode confirms that Lead **19** strikes an ideal balance between rigidity and longitudinal reach (*R8m*), occupying the hydrophobic mid-gorge without inducing steric strain.

In contrast, Compound **20** exhibited a distinct binding penalty that explains its marginally lower affinity. While it maintains the Asp74 H-bond in the PAS, the extra methylene unit in its *n*-pentyl chain introduces excessive conformational freedom and steric bulk. As highlighted in the docking snapshots (Figure 5, Row 4), Compound **20** suffers from an Unfavorable Acceptor-Acceptor interaction with Ser125 (indicated by the red bubble). Mechanistically, this electronic clash, combined with the loss of the critical π-π stacking contact with Trp86 in the CAS, suggests that the longer tail of **20** forces the aromatic core into a sub-optimal orientation. These findings corroborate the QSAR model’s prediction that structural complexity must be precisely tuned to the enzyme’s physical dimensions, positioning Lead **19** as the superior candidate for inhibiting both neurotransmitter hydrolysis and PAS-mediated Aβ peptide aggregation.

### 2.7. Molecular Dynamics (MD) Simulation and Dynamic Stability

**Figure 6:**
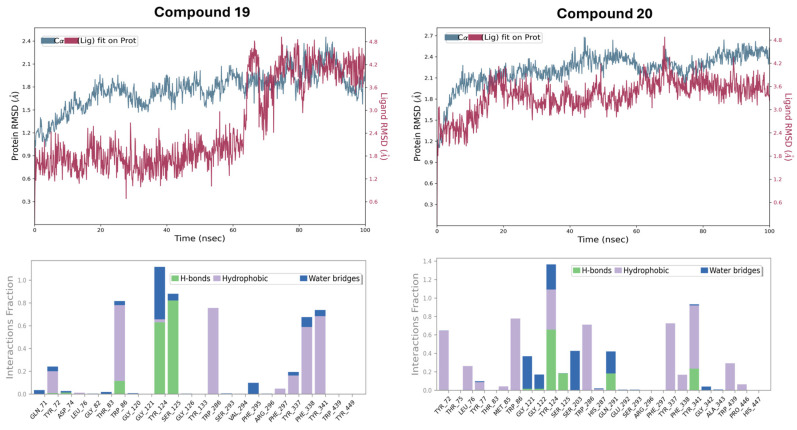
The 100 ns Molecular Dynamics (MD) simulation stability analysis: (top row) Protein-Ligand RMSD trajectories for Lead Compound **19** (left) and Compound **20** (right), illustrating the fitment adaptations and global stability; (bottom row) Protein-Ligand interaction fraction histograms detailing the occupancy of H-bonds, hydrophobic contacts, and water bridges across critical gorge residues (Tyr124, Ser125, Trp86, etc.).

To validate the thermodynamic stability of the docked poses and characterize the temporal nature of the ligand-enzyme interactions, 100 ns molecular dynamics (MD) simulations were executed. The protein-ligand root mean square deviation (PL-RMSD) served as the primary indicator of complex stability. For Lead Compound **19**, the protein backbone equilibrated within a range of 1.5–2.4 Å, signifying a stable global fold throughout the trajectory (Figure 6, top left). The ligand RMSD trajectory for Lead **19** exhibited a distinct conformational transition, maintaining a stable plateau at 1.8 Å until 64 ns, followed by a discrete shift to a new equilibrium at 4.2 Å. This 2.4 Å deviation represents a significant binding pose rearrangement within the 20-Å AChE gorge. Such a transition suggests that while the initial docked orientation was meta-stable, the dynamic environment of the solvated protein allowed the ligand to overcome a local energetic barrier and settle into a more robust, dynamically relaxed global minimum for the remainder of the simulation. This behavior underscores the importance of MD-based refinement over static docking for flexible, dual-site inhibitors.

In contrast, Compound **20** exhibited a more erratic RMSD profile, with protein fluctuations reaching ≈ 2.7 Å and a less stabilized ligand trajectory (Figure 6, top right). This relative loss of conformational stability is likely attributed to the entropic penalty and steric hindrance of the longer *n*-pentyl chain, which prevents the tricyclic core from achieving the high-occupancy anchoring observed for **19**.

The mechanistic basis for Lead **19**’s superior stability is elucidated by the protein-ligand interaction fraction histograms (Figure 6, bottom row). Compound **19** maintained high-occupancy contacts with critical active-site residues, specifically Tyr124, Ser125, and Trp86. The interaction fractions for Tyr124 and Ser125 exceeded 1.0, indicating the formation of multiple simultaneous contact types, including conventional hydrogen bonds and stable water-mediated bridges. Persistent hydrophobic interactions with Trp86, Phe338, and Tyr341 further anchored the molecule within the 20 Å gorge. For Compound **20**, while Asp74 and Trp286 interactions were maintained in the Peripheral Anionic Site (PAS), the occupancy of catalytic triad contacts was significantly reduced, corroborating the lower binding affinity reported in the docking studies.

The MD results systematically validate the GA-MLR model’s prioritization of Lead **19**. The ability of this sulfonamide-linked scaffold to maintain consistent engagement with both the CAS (Trp86) and PAS (Asp74, Tyr124) while undergoing adaptive fitment changes distinguishes it as a high-confidence lead for Alzheimer’s therapy. This data provided the essential dynamic proof that structural precision in the *R8m* (longitudinal reach) and *max_conj_path* (aromatic stacking) descriptors translates directly to target engagement under physiological conditions. While these 100 ns trajectories provide a robust basis for the comparative dynamic filtering of the prioritized leads, we recognize that a single simulation offers a focused view of the conformational ensemble. Consequently, the observed stability profiles are utilized here as a validation of the QSAR-derived binding hypotheses, with the understanding that future high-resolution mechanistic studies will benefit from multi-replicate sampling to further characterize long-term convergence.

### 2.8. Post-MD Integrity and Energy Landscapes

**Figure 7:**
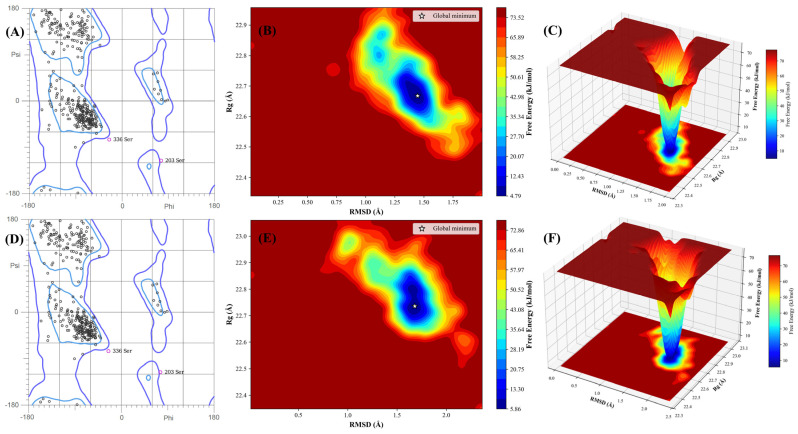
Integrated post-MD stereochemical and thermodynamic stability profiles for Lead Compounds **19** and **20.** (**A**–**C**) Lead **19**-AChE complex; (**D**–**F**) Compound **20**-AChE system. (**A**,**D**) Ramachandran plots confirming protein secondary structure integrity; black circles denote residues within favored regions (found in >89% of the protein) and pink circles identify outliers; (**B**,**E**) 2D Free Energy Landscapes highlighting global minima (1.680 Å, 22.736 Å, 5.859 kJ/mol for **19**; 1.446 Å, 22.668 Å, 4.794 kJ/mol for **20**); (**C**,**F**) 3D landscapes illustrating the superior thermodynamic convergence of Lead **19**.

The structural integrity of the human AChE enzyme following 100 ns ligand binding was rigorously validated via stereochemical analysis of the final trajectory frames. For both complexes, over 89.6% of residues were located within the favored regions of the Ramachandran plot, with a cumulative total of >97.9% in allowed regions. The minor population of outliers was strictly confined to intrinsically disordered surface loop regions, such as Ser67 and Ala141, confirming that the dual-site occupancy of the 20 Å gorge by our sulfonamide leads does not precipitate deleterious distortions in the protein backbone or catalytic machinery (Figure 7A,D).

**Figure 8:**
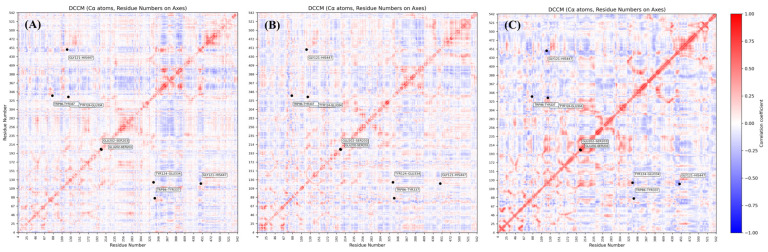
Dynamic Cross-Correlation Matrices (DCCM) illustrating the Cα residue-level synchrony over 100 ns. (**A**) Lead **19**-AChE complex; (**B**) Compound **20**-AChE complex; (**C**) Apo-enzyme baseline (blank) reference. The color scale indicates the degree of correlation (+1.0, red) or anti-correlation (−1.0, blue). Labeled residue pairs (Gly121-His447, Trp86-Tyr337, etc.) identify critical functional nodes where Lead **19** maintains native enzyme “breathing”, while Compound **20** introduces disruptive correlated rigidity.

The thermodynamic convergence of the binding states was evaluated by constructing the Free Energy Landscape (FEL) across the coordinates of global complex compactness (*Rg*) and RMSD. Lead Compound **19** achieved a highly stable binding state, reaching its global minimum at RMSD = 1.680 Å and *Rg* = 22.736 Å, with a relative conformational free energy (Δ*G*) of 5.859 kJ/mol (Figure 8B,C). The presence of a singular, deep energy funnel signifies that the n-butyl sulfonamide scaffold achieves a well-equilibrated, low-entropy binding mode.

In contrast, Compound **20** exhibited a more scattered energetic ensemble (Figure 7E,F). Its global minimum was identified at RMSD = 1.446 Å and *Rg* = 22.668 Å with a lower relative energy of 4.794 kJ/mol, yet the surrounding landscape is broader and less localized. This thermodynamic dispersion is a direct consequence of the increased conformational freedom of the n-pentyl chain, which prevents the tricyclic core from maintaining the rigid, high-occupancy orientation required for effective Aβ anti-aggregation through PAS blockade. These results systematically support the selection of Lead **19** as the superior candidate for clinical advancement.

### 2.9. Functional Residue Synchrony and Catalytic Integrity

The influence of ligand binding on the intrinsic global dynamics of human AChE was quantified using Dynamic Cross-Correlation Matrices (DCCM) for the Cα atoms (Figure 8). To establish a ground-truth for protein “breathing,” a ligand-free (Apo) simulation was utilized as a baseline reference (Figure 8C). In the Apo state, critical residue pairs such as Gly121-His447 (essential for the catalytic cycle) and Trp86-Tyr337 (responsible for substrate recognition) demonstrate pronounced anti-correlated motions (indicated by deep blue regions with *C_ij_* < −0.25).

Lead Compound **19** effectively preserves these native anti-correlations (Figure 8A), suggesting that its dual-site anchoring at the PAS and CAS does not perturb the essential flexibility required for catalytic throughput. Conversely, Compound **20** (Figure 8B) induces a deleterious transition toward correlated motions in the Trp86-Tyr337 pair and increased rigidity in the Tyr124-Glu334 loop. This disruption of native synchrony, combined with the scattered Free Energy Landscape (Figure 7), provides a strong computational rationale for prioritizing Lead **19** as a potential disease modifying candidate, pending future in vitro and in vivo experimental validation.

## 3. Materials and Methods

### 3.1. Dataset Curation and Structural Standardization

A primary dataset of 115 fused quinoline analogs with experimentally determined AChE inhibitory activities was curated from peer-reviewed medicinal chemistry literature. To ensure a rigorous and reproducible foundation, strict inclusion and exclusion criteria were applied: (1) structural homogeneity was maintained by focusing exclusively on quinoline and tacrine-based hybrids; (2) to minimize inter-laboratory experimental variability, only compounds evaluated via the standard or modified Ellman’s colorimetric assay (substrate: acetylthiocholine iodide; pH 8.0) were included. While the dataset incorporates IC_50_ values from multiple species (human, bovine, and electric eel), this is justified by the high evolutionary conservation of the 20-Å AChE active site gorge. The catalytic triad and peripheral anionic site (PAS) exhibit profound structural homology across these vertebrates, allowing 2D-topological and constitutional descriptors to capture the universal physicochemical requirements for dual-site inhibition.

To ensure data integrity, a rigorous deduplication protocol was enforced; where overlapping chemical structures were identified across different studies, the most recent or consistently reported value was retained to prevent statistical weighting bias. Biological activity values (IC_50_) were converted to the negative decadic logarithm (pIC_50_ = −log_10_ IC_50_ [M]) to ensure a normal distribution of data. Chemical structures were sketched in MarvinSketch (v24.3.1, ChemAxon), neutralized, and desalted. Initial 3D geometries were generated and subjected to energy minimization using the MMFF94 force field to a gradient convergence of 0.01 kcal/mol·Å to provide a consistent conformational starting point for descriptor calculation. A comprehensive list of all computational tools, software versions, and web servers utilized in this study is provided in Supplementary information (Table S1).

### 3.2. Descriptor Generation and Feature Selection

A comprehensive pool of 11,135 molecular descriptors, spanning constitutional, topological, electronic, and fragmental properties was generated via the Online Chemical Modeling Environment (OCHEM) web platform. Utilizing this open-access server ensures methodological reproducibility for all integrated descriptor libraries, including Dragon7 and alvaDesc [34]. To mitigate the curse of dimensionality and remove uninformative variables, a three-stage pre-filtering protocol was applied: (1) descriptors with near-constant values (>80% constant) were removed; (2) a pairwise correlation filter (|r| > 0.95) was applied to eliminate redundant variables; and (3) descriptors with low variance were excluded. This reduction yielded a refined pool of 2140 informative descriptors for subsequent GA-MLR model construction. 

### 3.3. QSAR Model Construction and Regulatory Validation

The QSAR was established using the Genetic Algorithm-Multiple Linear Regression (GA-MLR) technique within the QSARINS (v2.2.4) platform [35,36]. The primary curated dataset of 115 compounds was partitioned into a training set (*n* = 81) for feature selection and an internal test set (*n* = 34) to initially evaluate predictive capability. To rigorously verify the structural generalizability of the resulting model against unseen chemical space, a completely independent external validation set (*n* = 8) was subsequently curated from separate literature sources. The statistical robustness of the resulting model was evaluated against the guidelines established by the Organization for Economic Co-operation and Development (OECD). Predictive accuracy and internal stability were quantified using the coefficient of determination (*R*^2^), cross-validated *Q*^2^ (Leave-one-out and Leave-many-out), and the concordance correlation coefficient (CCC). The applicability domain (AD) was delineated through leverage analysis (Williams plot), with the leverage threshold (h*) calculated as 3(*p*’ + 1)/*n* (where *p*’ is the number of model descriptors and *n* is the training set size). Structural anchoring was confirmed by performing 2000 iterations of Y-randomization, ensuring that the biological endpoints were not derived from chance correlations.

### 3.4. Rational Design and ADMET Evaluation

The validated GA-MLR model served as the quantitative engine for the rational design of eighteen fused quinoline derivatives. Structural modifications were systematically guided by the prioritized descriptors *max_conj_path* (extended π-conjugation), *R8m* (longitudinal reach), and *MATS3s* (electronic distribution). The prospective library was structured to include sixteen novel scaffolds and two internal benchmarks, Compounds **2** and **4** from the external validation set, to serve as predictive calibration anchors. Pharmacokinetic and toxicological profiles were predicted using the ADMETlab 3.0 server to evaluate the clinical viability of the designed leads (Table S4) [37]. Critical parameters included aqueous solubility (logS) measured in log mol/L, Caco-2 permeability quantified as log cm/s to assess intestinal absorption, and a Blood–Brain Barrier (BBB) score threshold of >0.3 to confirm central nervous system accessibility. Synthetic accessibility was delineated on a 1–10 scale where 1 represents the highest ease of synthesis. Toxicological liabilities were quantified via the ProTox-II server to evaluate organ-specific hepatotoxicity and systemic probabilities for neurotoxicity, immunotoxicity, and cardiotoxicity relative to the reference drug Tacrine [38].

### 3.5. Molecular Docking and Molecular Dynamics Protocols

Molecular docking elucidated the binding orientations of prioritized Leads 19 and 20 within the human AChE crystal structure (PDB ID: 7XN1) at 2.85 Å resolution [39]. The protein was prepared by removing co-crystallized solvent and heteroatoms followed by the addition of polar hydrogens. The protein structure was processed using the CB-Dock2 cavity detection algorithm to automate the identification of optimal binding sites within the 20-Å enzyme gorge [40,41]. Scientific transparency was ensured by validating the docking protocol via self-docking of the co-crystallized ligand Tacrine; successful recovery of the experimental pose was defined by an RMSD of 0.326 Å, well within the 2.0 Å threshold for high-confidence binding mode prediction. Visualization of binding interfaces was conducted using ChimeraX [42] and ProteinPlus [43,44,45].

Long-range 100 ns MD simulations were conducted using the Desmond module in the Schrödinger Suite 2024-2 [46,47,48]. The lead-enzyme complexes were solvated in an orthorhombic box using the TIP3P water model with a 10 Å buffer. Systems were neutralized with Na^+^/Cl^−^ ions and subjected to the default Desmond relaxation protocol prior to production. Production runs were executed at 300 K and 1.01325 bar under the NPT ensemble utilizing the OPLS4 force field. To establish a comparative baseline for dynamic correlation analysis, a ligand-free apo-enzyme system was simulated utilizing the identical 100 ns protocol and thermodynamic parameters. Post-simulation analyses included Protein-Ligand RMSD, residue-level Root Mean Square Fluctuation (RMSF), and Radius of Gyration (rGyr) to evaluate temporal stability.

### 3.6. Post-Simulation Integrity and Functional Dynamics Analysis

The structural and thermodynamic consequences of ligand binding were evaluated using the MD-equilibrated trajectories. The stereochemical quality of the final 100 ns snapshots was verified through Ramachandran plot analysis using the MolProbity server, which quantified the distribution of (ϕ, Ψ) dihedral angles to ensure that binding within the narrow active-site gorge did not induce non-physical backbone strain [49]. Residues categorized as outliers were mapped back to the 3D structure to confirm their localization within flexible surface loops far from the binding interface.

The thermodynamic stability of the Lead 19 and Lead 20 complexes was further characterized by constructing the Free Energy Landscape (FEL). The FEL was generated by performing Principal Component Analysis (PCA) on the Cα coordinates to extract the primary modes of motion. The probability distribution of the trajectory across the first two principal components (PC1 and PC2) was used to calculate the relative Gibbs free energy (G) through the Boltzmann relationship, allowing for the identification of localized energy minima and the assessment of entropic instability in the ligand-bound states [50,51,52].

Functional synchrony between protein residues was quantified using the Dynamic Cross-Correlation Matrix (DCCM) [53]. Cross-correlation coefficients (*C_ij_*) were calculated for all Cα pairs throughout the 100 ns trajectory to evaluate how the inhibitors influenced the native “breathing” motions of the enzyme. Positive correlations (*C_ij_* > 0) denoted synchronized residue movement, while negative values (*C_ij_* < 0) identified anti-correlated motions. This analysis was critical for validating that Lead 19 preserves the essential anti-correlation of the Gly121-His447 pair required for the enzyme’s catalytic cycle.

## 4. Conclusions

This investigation successfully established a robust, statistically validated computational pipeline for the rational design of novel fused quinoline sulfonamides as dual-site acetylcholinesterase (AChE) inhibitors. By leveraging a five-descriptor GA-MLR QSAR model (*R*^2^ = 0.7569, $Q_{LOO}^{2}$ = 0.7244), we demonstrated that inhibitory potency is fundamentally governed by the extension of π-conjugation (*max_conj_path*) and the precise spatial mass distribution (*R8m*) required to bridge the ~20 Å enzyme gorge. Among the eighteen analogs prioritized, Lead Compound **19** emerged as a superior candidate, achieving high-occupancy dual-site anchoring at both the Catalytic (CAS) and Peripheral (PAS) sites while maintaining an optimized pharmacokinetic profile suitable for central nervous system penetration. In contrast, Compound **20** exhibited inferior thermodynamic stability and reduced binding efficiency, a direct consequence of an unfavorable stereoelectronic clash with Ser125 and the entropic instability introduced by its longer *n*-pentyl chain.

The hypothesized clinical potential of Lead **19** is further supported by its simulated ability to preserve the native functional synchrony of the enzyme, specifically the essential anti-correlated motions between the Gly121-His447 catalytic pair, while significantly reducing off-target risks for neurotoxicity and cardiotoxicity compared to the reference drug Tacrine. These findings provide a high-value structural scaffold to address the escalating global dementia crisis, particularly in high-burden demographic regions such as India. In alignment with the requirements of this Special Issue, this study provides the mechanistic and in silico foundation for the next phase of experimental validation. While biomimetic chromatography falls strictly outside the scope of the present purely computational study, this in silico framework provides the necessary structural foundation for future experimental validation using Immobilized Artificial Membrane (IAM) and Human Serum Albumin (HSA) stationary phases.

## Data Availability

The data supporting the results of this study are available within the article and its Supplementary Materials. Specifically, the curated dataset of 115 fused quinoline analogs, molecular descriptor values, and the coordinate files for the optimized GA-MLR model and 100 ns MD trajectories are available from the corresponding author upon reasonable request.

## Supplementary Materials

The following supporting information can be downloaded at: https://www.mdpi.com/article/10.3390/ijms27073286/s1.

## References

[1] Cummings J., Zhou Y., Lee G., Zhong K., Fonseca J., Cheng F. (2023). Alzheimer’s disease drug development pipeline: 2023. Alzheimer’s Dement..

[2] Xiaopeng Z., Jing Y., Xia L., Xingsheng W., Juan D., Yan L., Baoshan L. (2025). Global Burden of Alzheimer’s disease and other dementias in adults aged 65 years and older, 1991–2021: Population-based study. Front. Public Health.

[3] World Population Review Alzheimer’s Rates by Country 2026. https://worldpopulationreview.com/country-rankings/alzheimers-rates-by-country.

[4] Alzheimer’s Disease International Dementia Statistics. https://www.alzint.org/about/dementia-facts-figures/dementia-statistics/.

[5] Yiannopoulou K.G., Anastasiou A.I., Zachariou V., Pelidou S.-H. (2019). Reasons for Failed Trials of Disease-Modifying Treatments for Alzheimer Disease and Their Contribution in Recent Research. Biomedicines.

[6] Kong L., Yang X., Xu J. (2025). Comparison of safety of lecanemab and aducanumab: A real-world disproportionality analysis using the FDA adverse event reporting system. Front. Pharmacol..

[7] Hampel H., Elhage A., Cho M., Apostolova L.G., Nicoll J.A.R., Atri A. (2023). Amyloid-related imaging abnormalities (ARIA): Radiological, biological and clinical characteristics. Brain.

[8] Wu B., Hu Q., Tian F., Wu F., Li Y., Xu T. (2021). A pharmacovigilance study of association between proton pump inhibitor and dementia event based on FDA adverse event reporting system data. Sci. Rep..

[9] Saeed R., Tariq H.Z., Althobaiti A., Sadeghian N., Taslimi P., Al-Rashida M., Islam T., Thabet H.K., Aftab H., Şenol H. (2025). Design, synthesis, and multi-target evaluation of 4-phenyl quinoline-8-sulfonate thiosemicarbazones as potential anti-Alzheimer agents. Sci. Rep..

[10] Grabowska W., Bijak M., Szelenberger R., Gorniak L., Podogrocki M., Harmata P., Cichon N. (2025). Acetylcholinesterase as a Multifunctional Target in Amyloid-Driven Neurodegeneration: From Dual-Site Inhibitors to Anti-Agregation Strategies. Int. J. Mol. Sci..

[11] Cheng S., Song W., Yuan X., Xu Y. (2017). Gorge Motions of Acetylcholinesterase Revealed by Microsecond Molecular Dynamics Simulations. Sci. Rep..

[12] Rawat K., Tewari D., Bisht A., Chandra S., Tiruneh Y.K., Hassan H.M., Al-Emam A., Sindi E.R., Al-Dies A.-A.M. (2024). Identification of AChE targeted therapeutic compounds for Alzheimer’s disease: An in-silico study with DFT integration. Sci. Rep..

[13] Marucci G., Buccioni M., Dal Ben D., Lambertucci C., Volpini R., Amenta F. (2021). Efficacy of acetylcholinesterase inhibitors in Alzheimer’s disease. Neuropharmacology.

[14] Su T., Zhang T., Xie S., Yan J., Wu Y., Li X., Huang L., Luo H.-B. (2016). Discovery of novel PDE9 inhibitors capable of inhibiting Aβ aggregation as potential candidates for the treatment of Alzheimer’s disease. Sci. Rep..

[15] Zhang Y., Chen H., Li R., Sterling K., Song W. (2023). Amyloid β-based therapy for Alzheimer’s disease: Challenges, successes and future. Signal Transduct. Target. Ther..

[16] Hampel H., Hardy J., Blennow K., Chen C., Perry G., Kim S.H., Villemagne V.L., Aisen P., Vendruscolo M., Iwatsubo T. (2021). The Amyloid-β Pathway in Alzheimer’s Disease. Mol. Psychiatry.

[17] Luo J., Wärmländer S.K., Gräslund A., Abrahams J.P. (2016). Cross-interactions between the Alzheimer Disease Amyloid-β Peptide and Other Amyloid Proteins: A Further Aspect of the Amyloid Cascade Hypothesis. J. Biol. Chem..

[18] Goyal M., Dhanjal J.K., Goyal S., Tyagi C., Hamid R., Grover A. (2014). Development of dual inhibitors against Alzheimer’s disease using fragment-based QSAR and molecular docking. BioMed Res. Int..

[19] Ferreira J.P.S., Albuquerque H.M.T., Cardoso S.M., Silva A.M.S., Silva V.L.M. (2021). Dual-target compounds for Alzheimer’s disease: Natural and synthetic AChE and BACE-1 dual-inhibitors and their structure-activity relationship (SAR). Eur. J. Med. Chem..

[20] Grande G., Valletta M., Rizzuto D., Xia X., Qiu C., Orsini N., Dale M., Andersson S., Fredolini C., Laukka E.J. (2025). Blood-based biomarkers of Alzheimer’s disease and incident dementia in the community. Nat. Med..

[21] Qizilbash N., Birks J., Lopez Arrieta J., Lewington S., Szeto S. (2007). Withdrawn: Tacrine for Alzheimer’s disease. Cochrane Database Syst. Rev..

[22] Avram S., Mernea M., Limban C., Borcan F., Chifiriuc C. (2020). Potential Therapeutic Approaches to Alzheimer’s Disease By Bioinformatics, Cheminformatics and Predicted Adme-Tox Tools. Curr. Neuropharmacol..

[23] Nilewar S.S., Chobe S., Dudhe P., Kumar P.K., Lodha S., Raut A.D., Fernández-Conde D., Farhan M., Muteeb G., Pawar T.J. (2026). An Integrated QSAR-MD-DCCM Pipeline: A Predictive Computational Platform for the Rational Design and Dynamic Functional Validation of Dual-Target Directed Ligands. Pharmaceuticals.

[24] Moaddel R., Wainer I.W. (2006). Development of immobilized membrane-based affinity columns for use in the online characterization of membrane bound proteins and for targeted affinity isolations. Anal. Chim. Acta.

[25] Bastikar V., Bastikar A., Gupta P. (2022). Quantitative structure–activity relationship-based computational approaches. Computational Approaches for Novel Therapeutic and Diagnostic Designing to Mitigate SARS-CoV-2 Infection.

[26] Huang J., Fan X. (2011). Why QSAR Fails: An Empirical Evaluation Using Conventional Computational Approach. Mol. Pharm..

[27] Leardi R., Rogers J.P. (1996). Genetic Algorithms in Feature Selection. Principles of QSAR and Drug Design.

[28] Reddy A.S., Kumar S., Garg R. (2010). Hybrid-genetic algorithm based descriptor optimization and QSAR models for predicting the biological activity of Tipranavir analogs for HIV protease inhibition. J. Mol. Graph. Model..

[29] Gupta K., Kumar A., Patel R., Ghode P., Kumar H., Murmu A., Sahu N., Verma G., Sahu S., Soni S. (2025). Predictive QSAR Models Followed by Toxicity, Molecular Docking, and Molecular Dynamics Simulation in Search of Azole Derivatives as AChE Inhibitors for the Treatment of Alzheimer’s Disease. J. Chemom..

[30] Li Z.H., Yin L.Q., Zhao D.H., Jin L.H., Sun Y.J., Tan C. (2022). SAR studies of quinoline and derivatives as potential treatments for Alzheimer’s disease. Arab. J. Chem..

[31] Muñoz-Ruiz P., Rubio L., García-Palomero E., Dorronsoro I., Monte-Millán M.D., Valenzuela R., Usán P., de Austria C., Bartolini M., Andrisano V. (2005). Design, synthesis, and biological evaluation of dual binding site acetylcholinesterase inhibitors: New disease-modifying agents for Alzheimer’s disease. J. Med. Chem..

[32] Hariri R., Afshar Z., Mahdavi M., Safavi M., Saeedi M., Najafi Z., Sabourian R., Karimpour-Razkenari E., Edraki N., Moghadam F.H. (2016). Novel tacrine-based pyrano[3′,4′:5,6]pyrano[2,3-b]quinolinones: Synthesis and cholinesterase inhibitory activity. Arch. Pharm..

[33] da Costa J.S., Bizarro Lopes J.P., Russowsky D., Petzhold C.L., de Amorim Borges A.C., Ceschi M.A., Konrath E., Batassini C., Lunardi P.S., Saraiva Gonçalves C.A. (2013). Synthesis of tacrine-lophine hybrids via one-pot four component reaction and biological evaluation as acetyl- and butyrylcholinesterase inhibitors. Eur. J. Med. Chem..

[34] Sushko I., Pandey A.K., Novotarskyi S., Körner R., Rupp M., Teetz W., Brandmaier S., Abdelaziz A., Prokopenko V.V., Tanchuk V.Y. (2011). Online chemical modeling environment (OCHEM): Web platform for data storage, model development and publishing of chemical information. J. Cheminform..

[35] Gramatica P., Chirico N., Papa E., Cassani S., Kovarich S. (2013). QSARINS: A new software for the development, analysis, and validation of QSAR MLR models. J. Comput. Chem..

[36] Gramatica P., Cassani S., Chirico N. (2014). QSARINS-chem: Insubria datasets and new QSAR/QSPR models for environmental pollutants in QSARINS. J. Comput. Chem..

[37] Fu L., Shi S., Yi J., Wang N., He Y., Wu Z., Peng J., Deng Y., Wang W., Wu C. (2024). ADMETlab 3.0: An updated comprehensive online ADMET prediction platform enhanced with broader coverage, improved performance, API functionality and decision support. Nucleic Acids Res..

[38] Banerjee P., Kemmler E., Dunkel M., Preissner R. (2024). ProTox 3.0: A webserver for the prediction of toxicity of chemicals. Nucleic Acids Res..

[39] Dileep K., Ihara K., Mishima-Tsumagari C., Kukimoto-Niino M., Yonemochi M., Hanada K., Shirouzu M., Zhang K.Y. (2022). Crystal structure of human acetylcholinesterase in complex with tacrine: Implications for drug discovery. Int. J. Biol. Macromol..

[40] Yang X., Liu Y., Gan J., Xiao Z.-X., Cao Y. (2022). FitDock: Protein–ligand docking by template fitting. Brief. Bioinform..

[41] Liu Y., Yang X., Gan J., Chen S., Xiao Z.-X., Cao Y. (2022). CB-Dock2: Improved protein–ligand blind docking by integrating cavity detection, docking and homologous template fitting. Nucleic Acids Res..

[42] Meng E.C., Goddard T.D., Pettersen E.F., Ferrin T.E. (2023). UCSF ChimeraX: Tools for structure building and analysis. Protein Sci..

[43] Schöning-Stierand K., Diedrich K., Ehrt C., Flachsenberg F., Graef J., Sieg J., Penner P., Poppinga M., Ungethüm A., Rarey M. (2022). ProteinsPlus: A comprehensive collection of web-based molecular modeling tools. Nucleic Acids Res..

[44] Fährrolfes R., Bietz S., Flachsenberg F., Meyder A., Nittinger E., Rarey M. (2017). ProteinsPlus: A web portal for structure analysis of macromolecules. Nucleic Acids Res..

[45] Schöning-Stierand K., Diedrich K., Fährrolfes R., Flachsenberg F., Meyder A., Nittinger E., Steinegger R., Rarey M. (2020). ProteinsPlus: Interactive analysis of protein–ligand binding interfaces. Nucleic Acids Res..

[46] Bowers K.J., Chow D.E., Xu H., Dror R.O., Eastwood M.P., Gregersen B.A., Klepeis J.L., Kolossvary I., Moraes M.A., Sacerdoti F.D. (2006). Scalable Algorithms for Molecular Dynamics Simulations on Commodity Clusters. Proceedings of the 2006 ACM/IEEE Conference on Supercomputing (SC’06).

[47] Nilewar S.S., Chobe S.S., Gurav A.D., Kureshi S.B., Palande S.B., Escobar-Cabrera J., Hernández-Rosas F., Pawar T.J. (2026). Galloylation-Driven Anchoring of the Asp325-Asp336 Ridge: The Molecular Logic Behind the Superior Kinetic Stabilization of HMPV Fusion Protein by Green Tea Dimeric Catechins. Molecules.

[48] Michaud-Agrawal N., Denning E.J., Woolf T.B., Beckstein O. (2011). MDAnalysis: A toolkit for the analysis of molecular dynamics simulations. J. Comput. Chem..

[49] Williams C.J., Headd J.J., Moriarty N.W., Prisant M.G., Videau L.L., Deis L.N., Verma V., Keedy D.A., Hintze B.J., Chen V.B. (2018). MolProbity: More and better reference data for improved all-atom structure validation. Protein Sci..

[50] Hunter J.D. (2007). Matplotlib: A 2D Graphics Environment. Comput. Sci. Eng..

[51] Virtanen P., Gommers R., Oliphant T.E., Haberland M., Reddy T., Cournapeau D., Burovski E., Peterson P., Weckesser W., Bright J. (2020). SciPy 1.0: Fundamental algorithms for scientific computing in Python. Nat. Methods.

[52] Harris C.R., Millman K.J., van der Walt S.J., Gommers R., Virtanen P., Cournapeau D., Wieser E., Taylor J., Berg S., Smith N.J. (2020). Array programming with NumPy. Nature.

[53] Ichiye T., Karplus M. (1991). Collective motions in proteins: A covariance analysis of atomic fluctuations in molecular dynamics and normal mode simulations. Proteins.

