# Supplementary material for: Dual-Site Acetylcholinesterase Inhibition and Multiscale Stability of Fused Quinoline Sulfonamides: A Chemoinformatic GA-MLR and Molecular Dynamics Study
Source: Int J Mol Sci. 2026 Apr 4;27(7):3286. doi: 10.3390/ijms27073286 (PMC13074083; doi:10.3390/ijms27073286)
Supplement: Supplementary file 1 [file ijms-27-03286-s001.zip › ijms-4215546-supplementary.pdf]

Supplementary Information

Dual-Site Acetylcholinesterase Inhibition and Multiscale Stability of Fused Quinoline Sulfonamides: A Chemoinformatic GA-MLR and Molecular Dynamics Study

Shrikant S. Nilewar [1], Apurva D. Chavan [2], Ankita R. Pradhan [2], Anshuman A. Tripathy [2], Sandesh Lodha [1], Nagaraju Bandaru [3], Prashik B. Dudhe [2], Perli Kranti Kumar [4], Ghazala Muteeb [5], Antonio Jose Naranjo-Redondo [6,] and Tushar Janardan Pawar [7,]**

[1] *Department of Pharmaceutical Chemistry, Maliba Pharmacy College, Uka Tarsadia University, Bardoli, 394350, Gujrat, India; (S.S.N.) shrinilewar@gmail.com; (S.L.) sandeshlodha@gmail.com*

[2] *School of Pharmaceutical Science, Sandip University, Nashik, 422213, Maharashtra, India; (A.D.C) apurvachavan237@gmail.com; (A.R.P.) ankitap41103@gmail.com; (A.A.T.) tripathyanshuman42@gmail.com*

[3] *Department of Pharmacology, Sree Dattha Institute of Pharmacy Sheriguda, Ibrahimpatnam, Hyderabad, 501510, Telangana, India; bnrajupharma@gmail.com*

[4] *Department of Pharmaceutical Analysis, J.K.K. Nattraja College of Pharmacy, Kumarapalayam, 638183, Tamil Nadu, India; dudhe.prashik@gmail.com*

[5] *Department of Nursing, College of Applied Medical Sciences, King Faisal University, Al-Ahsa, Saudi Arabia. graza@kfu.edu.sa*

[6] *División de Ingeniería, Universidad Anáhuac Querétaro, Circuito Universidades I, Fracción 2 S/N, Zibatá, El Marqués, 76246, Querétaro, Mexico; antonio.naranjo@anahuac.mx*

[6] *Centro de Investigación, Universidad Anáhuac Querétaro, Circuito Universidades I, Fracción 2 S/N, Zibatá, El Marqués, 76246, Querétaro, Mexico; tushar.pawar@anahuac.mx*

* *Correspondence: (A.J.N.R.) antonio.naranjo@anahuac.mx; (T.J.P.) tushar.pawar@anahuac.mx*

S1. Computational Tools, Software and workflow

Table S1: Comprehensive List of Computational Tools and Software

Sr. No	Tools	Application	Web link
1	ChEMBL	Literature review and structure retrieval	https://www.ebi.ac.uk/chembl/
2	Bindingdb		https://www.bindingdb.org/
3	QSARINS	QSAR modelling	https://dunant.dista.uninsubria.it/qsar/?page_id=460
4	Marvin sketch	Ligand sketching and optimization	https://chemaxon.com/marvin
5	RCSB:PDB	Protein database	https://www.rcsb.org/
6	CB-Dock2	Protein-ligand docking tool	http://cadd.labshare.cn/cb-dock2/index.php
7	AutoDock Vina	Docking module used in CB-Dock2	https://vina.scripps.edu/
8	DockRMSD	Docking validation	https://zhanggroup.org/DockRMSD/
9	Discovery studio visualizer	2D interaction analysis	https://discover.3ds.com/discovery-studiovisualizer-download
10	Swisspdb Viewer	Preparation of Protein	https://spdbv.unil.ch/contactSurf_tut.html
11	Protein Plus	3D binding pocket visualizer	https://proteins.plus/
12	ADMETlab 3.0	ADMET profiling of the candidates	https://admetlab3.scbdd.com/
13	ProTox	Toxicity profiling	https://tox.charite.de/protox3/
14	Desmond/Schrodinger Suit	Molecular dynamic simulation studies	https://www.schrodinger.com/products/maestro
15	Ramachandran Plot	Post_MD stability analysis	https://molprobity.biochem.duke.edu/index.php
16	MD TASK	Dynamic cross correlation matrix	https://bioinformaticshome.com/db/tool/MD-TASK

QSAR Descriptor Definitions

Table S2: Definitions and Mechanistic Interpretation of Key Molecular Descriptors.

Descriptor	Full Definition and Origin	Mechanistic Interpretation in Models
**max_conj_path**	**Length of Conjugation.** A constitutional descriptor that measures the length of the longest conjugated system inside a molecule.	Its positive correlation implies that extended pi-pi interactions with aromatic residues in the AChE active site, such as Trp86 and Phe338, are facilitated by extended conjugation, frequently found in fused aromatic systems like quinoline derivatives.
**MATS3s**	**Electronic Distribution.** A 2D-autocorrelation measure weighted by intrinsic state values at a topological lag of 3.	Its high coefficient indicates that specific electronic environments distributed across the molecular graph are crucial for establishing favorable electrostatic and pi-cation interactions within the AChE gorge.
**R8m**	**Topological Ring Interactions.** A spatial descriptor representing ring interactions at a topological distance of 8.	Highlights the significance of extended or bridged ring systems. It describes how the fused-ring quinoline scaffold can effectively align, reach across, and anchor onto multiple subsites within the narrow and elongated AChE binding pocket.
**C-N-C=O**	**Functional Group Presence.** A fragment-based descriptor (derived from Fragmentor) indicating the presence of functional groups similar to carbamoyl or urea.	These groups act as essential hydrogen bond acceptors or donors, which is an important characteristic for interacting with critical residues like Glu202 or Ser203 in the catalytic triad of AChE.
**MNA -H(-C(C-H-H-C))**	**Substructural Environment.** A Multilevel Neighborhoods of Atoms (MNA) descriptor representing a specific hydrogen-centered substructure in a methyl-bearing environment.	Suggests that small steric or electronic effects within these specific local environments play a significant role in modulating binding affinity through targeted van der Waals or hydrophobic contacts.

Full Dataset:

Table S3: 115 original molecules used for model training and validation. SMILES, the experimental IC_{50}, and the calculated pIC_{50} value.

ID	Name	Activity/Expt PIC50	Status	Pred. by model eq.
1	NC1=C2CC3=C(C=CC=C3Br)C2=NC2=CC=CC=C12	6.638	Training	6.3757
2	NC1=C2CC3=C(C=CC=C3C3=CC=CC=C3)C2=NC2=CC=CC=C12	7.431	Training	6.7565
3	CC1(C)C\C(=N/NC(N)=N)C2=C(C1)N=C1C=C3OCOC3=CC1=C2	5.07	Training	5.2873
4	NC1=C2CC3=C(C=CC(=C3)C3=CC=CC=C3)C2=NC2=CC=CC=C12	7.244	Prediction	6.6416
5	NC1=C2CC3=C(C=C(C=C3)C3=CC=CC=C3)C2=NC2=CC=CC=C12	7.251	Training	6.6758
6	C[C@@H]1CC(=O)OC2=C1C=CC(OC1=CC=C(NC3=C4CCCC4=NC4=CC(Cl)=CC=C34)C=C1)=C2	4.786	Prediction	5.2919
7	C[C@@H]1CC(=O)OC2=C1C=CC(OC1=CC=C(NC3=C4CCCCC4=NC4=CC(Cl)=CC=C34)C=C1)=C2	4.718	Training	5.5704
8	ClC1=C(Cl)N=CC(=C1)C(=O)NCCCCCCNC1=C2CCCC2=NC2=CC=CC=C12	7.276	Training	7.8217
9	ClC1=CC=C(C=N1)C(=O)NCCCCCCNC1=C2CCCC2=NC2=CC=CC=C12	7.173	Training	7.4614
10	ClC1=CC=C(C=N1)C(=O)NCCCCCCCCNC1=C2CCCC2=NC2=CC=CC=C12	7.137	Prediction	7.5266
11	CC1=CC(=O)OC2=CC(OC3=CC=C(NC4=C5CCCCC5=NC5=CC(Cl)=CC=C45)C=C3)=CC=C12	4.743	Training	5.7161
12	ClC1=C(Cl)N=CC(=C1)C(=O)NCCCCCCCNC1=C2CCCC2=NC2=CC=CC=C12	6.903	Prediction	7.7856
13	CC(C)(C)C1=CC(CNCCNC2=C3CCCCCC3=NC3=CC=CC=C23)=CC(=C1O)C(C)(C)C	5.722	Training	5.6626
14	CC(C)(C)C1=CC(\C=N\CCNC2=C3CCCCCC3=NC3=CC=CC=C23)=CC(=C1O)C(C)(C)C	5.393	Prediction	5.6921
15	CC(C)(C)C1=CC(CNCCNC2=C3CCCC3=NC3=CC=CC=C23)=CC(=C1O)C(C)(C)C	5.457	Training	5.5676
16	CC1=CC=C(C=C1)S(=O)(=O)NCCCCNC1=C2CCCCC2=NC2=CC=CC=C12	6.176	Training	5.9924

17	CC1=CC=C(C=C1)S(=O)(=O)NCCCCCNC1=C2CCCCC2=NC2=CC=CC=C12	6.882	Training	5.9931
18	O=C(NCCNC1=C2CCCCC2=NC2=CC=CC=C12)\C=C\C1=CC=C(OCC2=CC=CC=C2)C=C1	7.218	Prediction	6.8324
19	COC1=CC=C(CN2C=C(C(=O)NCCCCCCNC3=C4CCCCC4=NC4=CC=C(OC)C=C34)C(=O)C3=CC=CC=C23)C=C1	5.814	Prediction	7.6226
20	COC1=CC=C(CN2C=C(C(=O)NCCCNC3=C4CCCCC4=NC4=CC=CC=C34)C(=O)C3=CC=CC=C23)C=C1	6.888	Training	7.5555
21	COC1=C(OCC2=CC=CC=C2)C=CC(\C=C\C(=O)NCCNC2=C3CCCCC3=NC3=CC=CC=C23)=C1	7.257	Training	7.1816
22	COC1=CC=C(CN2C=C(C(=O)NCCCNC3=C4CCCCC4=NC4=CC(Cl)=CC=C34)C(=O)C3=CC=CC=C23)C=C1	8.826	Training	7.526
23	COC1=CC(\C=C\C(=O)NCCNC2=C3CCCCC3=NC3=CC=CC=C23)=CC=C1OCC1=CC=C(Br)C=C1	7.305	Training	7.3928
24	COC1=CC(\C=C\C(=O)NCCNC2=C3CCCCC3=NC3=CC=CC=C23)=CC=C1OCC1=CC=C(C)C(C)=C1	7.431	Training	7.2556
25	COC1=CC=C(CNC(=O)CC2=CN(CCNC3=C4CCCCC4=NC4=CC(Cl)=CC=C34)N=N2)C=C1C	8	Training	8.0066
26	S=C(NCCCCNC1=C2CCCCC2=NC2=CC=CC=C12)NCCC1=CNC2=C1C=CC=C2	7.602	Training	7.4712
27	CCOP(=O)(NCCCCCCNC1=C2CCCCC2=NC2=CC=CC=C12)OCC	5.82	Training	5.6544
28	O=P(NCCCCCCNC1=C2CCCCC2=NC2=CC=CC=C12)(OC1=CC=CC=C1)OC1=CC=CC=C1	6.104	Training	6.0593
29	COC1=CC(\C=C\C(=O)NCCNC2=C3CCCCC3=NC3=CC=CC=C23)=CC=C1OCC1=CC=C(C=C1)C(F)(F)F	6.889	Training	7.3804
30	OCCC1=CN(CCNC2=C3CCCCC3=NC3=CC(Cl)=CC=C23)N=N1	6.698	Training	7.1321
31	CON1C=C(CCNC(=S)NCCCCCNC2=C3CCCCC3=NC3=CC=CC=C23)C2=C1C=CC=C2	7.155	Training	7.5375
32	CCCCOP(=O)(NCCCCCCCCNC1=C2CCCCC2=NC2=CC=CC=C12)OCCCC	6.038	Training	5.9535

33	O=P(NCCCCCCCCCCCCNC1=C2CCCCC2=NC2=CC=CC=C12)(OC1=CC=CC=C1)OC1=CC=CC=C1	6.001	Prediction	6.1412
34	COC1=CC=C(C=C1)[C@H]1C2=C(C)NN=C2OC2=C1C(N)=C1CCCCC1=N2	6.51	Training	5.8324
35	COC1=C(OC)C=C(C=C1)[C@H]1C2=C(C)NN=C2OC2=C1C(N)=C1CCCCC1=N2	6.721	Prediction	6.3178
36	CCCC1=NNC2=C1[C@H](C1=CC=C(OC)C=C1)C1=C(O2)N=C2CCCCC2=C1N	7.282	Training	7.5979
37	COC1=CC=C(C=C1)[C@H]1C2=C(NN=C2C2=CC=CC=C2)OC2=C1C(N)=C1CCCCC1=N2	7.089	Prediction	6.4807
38	CC1=NN(C2=C1[C@H](C1=CC=CC=C1)C1=C(O2)N=C2CCCCC2=C1N)C1=CC=CC=C1	5.558	Training	6.2623
39	COC1=CC=C(C=C1)[C@H]1C2=C(OC3=C1C(N)=C1CCCCC1=N3)N(N=C2C)C1=CC=CC=C1	5.548	Prediction	6.2959
40	NC1=C2CCCCC2=NC2=C1[C@H](C1=C(O2)N(N=C1C1=CC=CO1)C1=CC=CC=C1)C1=CC=CC=C1	7.143	Training	7.1641
41	NC1=C2CCCCC2=NC2=C1[C@@H](C1=CC=CC=C1)C1=C(O2)C2=C(C=CC=C2)C=C1	6.522	Prediction	6.3103
42	CC1=CC=C(C=C1)[C@H]1C2=C(OC3=C1C(N)=C1CCCCC1=N3)C1=C(C=CC=C1)C=C2	6.398	Training	6.222
43	COC1=CC=C([C@H]2C3=C(OC4=C2C(N)=C2CCCCC2=N4)C2=C(C=CC=C2)C=C3)C(OC)=C1	6.431	Training	6.304
44	COC1=CC(=CC=C1O)[C@H]1C2=C(OC3=C1C(N)=C1CCCCC1=N3)C1=C(C=CC=C1)C=C2	6.481	Training	6.7938
45	CCOC(=O)C1=C(OC2=NC3=C(CCCC3)C(N)=C2[C@H]1C1=CC=CC=C1Cl)C1=CC=CC=C1	6.814	Training	6.4856
46	CCOC(=O)C1=C(OC2=NC3=C(CCCC3)C(N)=C2[C@H]1C1=CC=CC(Cl)=C1)C1=CC=CC=C1	6.642	Training	6.4818
47	NC1=C2[C@@H](C3=CC=C(F)C=C3)C3=C(OC2=NC2=C1CCCC2)C(=O)C1=C(C=CC=C1)C3=O	6.051	Training	6.6577
48	CCOC(=O)C1=C(OC2=NC3=C(CCCC3)C(N)=C2[C@H]1C1=C(Br)C=CC=C1)C1=CC=CC=C1	6.927	Training	6.9654

49	CCOC(=O)C1=C(OC2=NC3=C(CCCC3)C(N)=C2[C@H]1C1=CC=CC(Br)=C1)C1=CC=CC=C1	7.16	Prediction	6.9577
50	O=C(CCC1=CNC2=C1C=CC=C2)NCCCCCNC1=C2CCCCC2=NC2=CC=CC=C12	7.155	Training	8.2841
51	ClC1=CC=C2C(NCCCCCNC(=O)CCC3=CNC4=C3C=CC=C4)=C3CCCCC3=NC2=C1	8.398	Prediction	8.4237
52	ClC1=CC=C2C(NCCCCCCCNC(=O)CCC3=CNC4=C3C=CC=C4)=C3CCCCC3=NC2=C1	10.223	Training	8.5042
53	ClC1=CC=C2C(NCCCCCCCCNC(=O)CCC3=CNC4=C3C=CC=C4)=C3CCCCC3=NC2=C1	9.301	Training	8.5627
54	ClC1=CC=C2C(NCCCCCCCCCNC(=O)CCC3=CNC4=C3C=CC=C4)=C3CCCCC3=NC2=C1	8.356	Prediction	8.5369
55	ClC1=CC=C2C(NCCCCCCCCCNC(=O)CCC3=CNC4=C3C=CC=C4)=C3CCCCC3=NC2=C1	7.658	Training	8.5369
56	CN(CCCNC(=O)CCC1=CNC2=C1C=CC=C2)CCCNC1=C2CCCCC2=NC2=CC=CC=C12	6.832	Training	8.2542
57	CN(CCCNC(=O)CCC1=CNC2=C1C=CC=C2)CCCNC1=C2CCCCC2=NC2=CC(Cl)=CC=C12	8.538	Training	8.4168
58	ClC1=CC=C2C(NCCCCCCNC(=O)CCC3=CNC4=C3C=C(C=C4)C#N)=C3CCCCC3=NC2=C1	9.155	Prediction	8.6035
59	ClC1=CC=C2C(NCCCCCCNC(=O)\C=C\C3=CNC4=C3C=CC=C4)=C3CCCCC3=NC2=C1	7.744	Prediction	7.3218
60	ClC1=CC=C2C(NCCCCCNC(=O)C3=CNC4=C3C=CC=C4)=C3CCCCC3=NC2=C1	6.745	Training	7.2884
61	ClC1=CC=C2C(NCCCCCCNC(=O)C3=CNC4=C3C=CC=C4)=C3CCCCC3=NC2=C1	7.481	Training	7.3984
62	ClC1=CC=C2C(NCCCCCCCNC(=O)C3=CNC4=C3C=CC=C4)=C3CCCCC3=NC2=C1	7.444	Prediction	7.3656
63	ClC1=CC=C2C(NCCCCCCCCNC(=O)C3=CNC4=C3C=CC=C4)=C3CCCCC3=NC2=C1	7.336	Training	7.4446

64	ClC1=CC=C2C(NCCCCCCCNC(=O)CC3=CNC4=C3C=CC=C4)=C3CCCCC3=NC2=C1	9.698	Training	8.5325
65	ClC1=CC=C2C(NCCCCCCCNC(=O)CC3=CNC4=C3C=C(Br)C=C4)=C3CCCCC3=NC2=C1	9.522	Prediction	8.5139
66	ClC1=CC=C2C(NCCCCCNC(=O)CCCC3=CNC4=C3C=CC=C4)=C3CCCCC3=NC2=C1	9.221	Training	8.3881
67	ClC1=CC=C2C(NCCCCCCNC(=O)CCCC3=CNC4=C3C=CC=C4)=C3CCCCC3=NC2=C1	9.301	Training	8.4785
68	CN(CCCCCCCNC1=C2CCCCC2=NC2=CC(Cl)=CC=C12)C(=O)C1=CNC2=C1C=CC=C2	8.962	Training	8.7358
69	ClC1=CC=C2C(=C1)N=C1CCCCC1=C2NCCCCCCCNC(=O)C1=NNC2=C1C=CC=C2	7.973	Training	7.6793
70	ClC1=CC=C2C(NCCCCCNC(=O)OCCC3=CNC4=C3C=CC=C4)=C3CCCCC3=NC2=C1	8.823	Training	8.5927
71	ClC1=CC=C2C(NCCCCCCNC(=O)OCCC3=CNC4=C3C=CC=C4)=C3CCCCC3=NC2=C1	9.155	Training	8.5477
72	ClC1=CC=C2C(NCCCCCCCNC(=O)OCCC3=CNC4=C3C=CC=C4)=C3CCCCC3=NC2=C1	8.522	Training	8.6116
73	ClC1=CC=C(C=C1)C1=C2CCCOC2=C2C=C(C=CC2=N1)C(=O)NCCCCCCNC1=C2CCCCC2=NC2=CC(Cl)=CC=C12	7.715	Training	7.9761
74	ClC1=CC=C(C=C1)C1=C2CCCOC2=C2C=C(C=CC2=N1)C(=O)NCCCCCCCNC1=C2CCCCC2=NC2=CC(Cl)=CC=C12	7.736	Training	8.0359
75	ClC1=CC=C(C=C1)C1=C2CCCOC2=C2C=C(C=CC2=N1)C(=O)NCCCCCCCCNC1=C2CCCCC2=NC2=CC(Cl)=CC=C12	8.553	Training	8.1271
76	ClC1=CC=C(C=C1)C1=C2CCCOC2=C2C=C(C=CC2=N1)C(=O)NCCCCCCCCCNC1=C2CCCCC2=NC2=CC(Cl)=CC=C12	7.604	Prediction	8.0775
77	ClC1=CC=C(C=C1)C1=C2CCCOC2=C2C=C(C=CC2=N1)C(=O)NCCCCCCCCCCNC1=C2CCCCC2=NC2=CC(Cl)=CC=C12	7.301	Prediction	8.1286

78	ClC1=CC=C2C(=C1)N=C1CCCCC1=C2NCCCCNC(=O)CCC1=CC2=C3OCCCC3=C(N=C2C=C1)C1=CC=CC=C1	7.78	Training	8.8122
79	ClC1=CC=C2C(=C1)N=C1CCCCC1=C2NCCCCCNC(=O)CCC1=CC2=C3OCCCC3=C(N=C2C=C1)C1=CC=CC=C1	8.416	Training	8.9242
80	ClC1=CC=C2C(=C1)N=C1CCCCC1=C2NCCCCCCNC(=O)CCC1=CC2=C3OCCCC3=C(N=C2C=C1)C1=CC=CC=C1	8.453	Prediction	8.8527
81	ClC1=CC=C2C(=C1)N=C1CCCCC1=C2NCCCCCCCNC(=O)CCC1=CC2=C3OCCCC3=C(N=C2C=C1)C1=CC=CC=C1	8.84	Training	8.877
82	ClC1=CC=C2C(=C1)N=C1CCCCC1=C2NCCCCCCCCNC(=O)CCC1=CC2=C3OCCCC3=C(N=C2C=C1)C1=CC=CC=C1	8.853	Training	8.9845
83	CN1C2=CC=CC=C2[C@H](NCCCCCCC(=O)NCCNC2=C3CCCCC3=NC3=CC=CC=C23)C2=CC=C(Cl)C=C2S1(=O)=O	7.085	Training	7.3397
84	CN1C2=CC=CC=C2[C@H](NCCCCCCC(=O)NCCNC2=C3CCCCC3=NC3=CC(Cl)=CC=C23)C2=CC=C(Cl)C=C2S1(=O)=O	8.497	Training	7.4638
85	CN1C2=CC=CC=C2[C@H](NCCCCCCC(=O)NCCCNC2=C3CCCCC3=NC3=CC=CC=C23)C2=CC=C(Cl)C=C2S1(=O)=O	7.276	Prediction	7.4361
86	CN1C2=CC=CC=C2[C@H](NCCCCCCC(=O)NCCCNC2=C3CCCCC3=NC3=CC(Cl)=CC=C23)C2=CC=C(Cl)C=C2S1(=O)=O	8.567	Training	7.4003
87	CN1C2=CC=CC=C2[C@H](NCCCCCCC(=O)NCCCCNC2=C3CCCCC3=NC3=CC=CC=C23)C2=CC=C(Cl)C=C2S1(=O)=O	6.809	Training	7.4268
88	CN1C2=CC=CC=C2[C@H](NCCCCCCC(=O)NCCCCNC2=C3CCCCC3=NC3=CC(Cl)=CC=C23)C2=CC=C(Cl)C=C2S1(=O)=O	7.436	Training	7.4012
89	CN1C2=CC=CC=C2[C@H](NCCCCCCC(=O)NCCCCCNC2=C3CCCCC3=NC3=CC=CC=C23)C2=CC=C(Cl)C=C2S1(=O)=O	6.967	Training	7.4645
90	CN1C2=CC=CC=C2[C@H](NCCCCCCC(=O)NCCCCCNC2=C3CCCCC3=NC3=CC(Cl)=CC=C23)C2=CC=C(Cl)C=C2S1(=O)=O	7.549	Training	7.4422
91	CN1C2=CC=CC=C2[C@H](NCCCCCCC(=O)NCCCCCCNC2=C3CCCCC3=NC3=	6.934	Prediction	7.3573

	CC=CC=C23)C2=CC=C(Cl)C=C2S1(=O)=O			
92	CN1C2=CC=CC=C2[C@H](NCCCCCCC(=O)NCCCCCCNC2=C3CCCCC3=NC3=CC(Cl)=CC=C23)C2=CC=C(Cl)C=C2S1(=O)=O	7.407	Training	7.4554
93	CN1C2=CC=CC=C2[C@H](NCCCCCCC(=O)NCCCCCCCNC2=C3CCCCC3=NC3=CC=CC=C23)C2=CC=C(Cl)C=C2S1(=O)=O	7.187	Prediction	7.3774
94	CN1C2=CC=CC=C2[C@H](NCCCCCCC(=O)NCCCCCCCNC2=C3CCCCC3=NC3=CC(Cl)=CC=C23)C2=CC=C(Cl)C=C2S1(=O)=O	6.879	Prediction	7.3808
95	CN1C2=CC=CC=C2[C@H](NCCCCCCC(=O)NCCCCCCCCNC2=C3CCCCC3=NC3=CC=CC=C23)C2=CC=C(Cl)C=C2S1(=O)=O	7.06	Training	7.4253
96	CN1C2=CC=CC=C2[C@H](NCCCCCCC(=O)NCCCCCCCCNC2=C3CCCCC3=NC3=CC(Cl)=CC=C23)C2=CC=C(Cl)C=C2S1(=O)=O	6.346	Training	7.4172
97	ClC1=CC=C(C=C1)C1=NC(=C(N1CCCCCCCCNC1=C2CCCCC2=NC2=CC=CC=C12)C1=CC=CC=C1)C1=CC=CC=C1	8.231	Prediction	7.5763
98	FC1=CC=C(C=C1)C1=NC(=C(N1CCCCCCCCNC1=C2CCCCC2=NC2=CC=CC=C12)C1=CC=CC=C1)C1=CC=CC=C1	7.586	Training	7.1117
99	COC1=CC=C(C=C1)C1=NC(=C(N1CCCCCCCCNC1=C2CCCCC2=NC2=CC=CC=C12)C1=CC=CC=C1)C1=CC=CC=C1	6.9	Prediction	7.6429
100	C(CCCNC1=C2CCCCC2=NC2=CC=CC=C12)CCCN1C(=NC(=C1C1=CC=CC=C1)C1=CC=CC=C1)C1=CC=CC=C1	7.094	Prediction	7.677
101	ClC1=CC=C(C=C1)C1=NC(=C(N1CCCCCCCNC1=C2CCCCC2=NC2=CC=CC=C12)C1=CC=CC=C1)C1=CC=CC=C1	7.221	Training	7.5405
102	FC1=CC=C(C=C1)C1=NC(=C(N1CCCCCCCNC1=C2CCCCC2=NC2=CC=CC=C12)C1=CC=CC=C1)C1=CC=CC=C1	7.413	Training	7.1993
103	COC1=CC=C(C=C1)C1=NC(=C(N1CCCCCCCNC1=C2CCCCC2=NC2=CC=CC=C12)C1=CC=CC=C1)C1=CC=CC=C1	7.161	Training	7.5235
104	CC1=CC2=C([C@@H](C3=CC=CC=C3)C3=C(O2)N=C2CCCCC2=C3N)C(=O)O1	5.711	Training	5.4429

105	CC1=CC2=C([C@@H](C3=CC=CC=C3F)C3=C(O2)N=C2CCCCC2=C3N)C(=O)O1	5.726	Prediction	5.5962
106	CC1=CC2=C([C@@H](C3=CC=C(F)C=C3)C3=C(O2)N=C2CCCCC2=C3N)C(=O)O1	5.387	Prediction	5.7443
107	CC1=CC2=C([C@@H](C3=CC=CC=C3Cl)C3=C(O2)N=C2CCCCC2=C3N)C(=O)O1	6.096	Prediction	5.6449
108	CC1=CC2=C([C@@H](C3=CC=C(Cl)C=C3)C3=C(O2)N=C2CCCCC2=C3N)C(=O)O1	6.086	Training	5.9946
109	CC1=CC2=C([C@@H](C3=CC=CC(Cl)=C3Cl)C3=C(O2)N=C2CCCCC2=C3N)C(=O)O1	6.431	Training	6.1227
110	CC1=CC2=C([C@@H](C3=CC=CC=C3C)C3=C(O2)N=C2CCCCC2=C3N)C(=O)O1	5.705	Prediction	5.4649
111	CC1=CC2=C([C@@H](C3=CC=CC(C)=C3)C3=C(O2)N=C2CCCCC2=C3N)C(=O)O1	5.93	Prediction	5.3996
112	CC1=CC2=C([C@@H](C3=CC=C(C)C=C3)C3=C(O2)N=C2CCCCC2=C3N)C(=O)O1	5.949	Training	5.4773
113	COC1=CC=C(C=C1)[C@@H]1C2=C(OC3=C1C(N)=C1CCCCC1=N3)C=C(C)OC2=O	5.705	Training	5.5435
114	COC1=CC(=CC(OC)=C1OC)[C@@H]1C2=C(OC3=C1C(N)=C1CCCCC1=N3)C=C(C)OC2=O	5.527	Training	6.0185
115	CC1=CC2=C([C@@H](C3=CC=CS3)C3=C(O2)N=C2CCCCC2=C3N)C(=O)O1	5.277	Training	5.4358

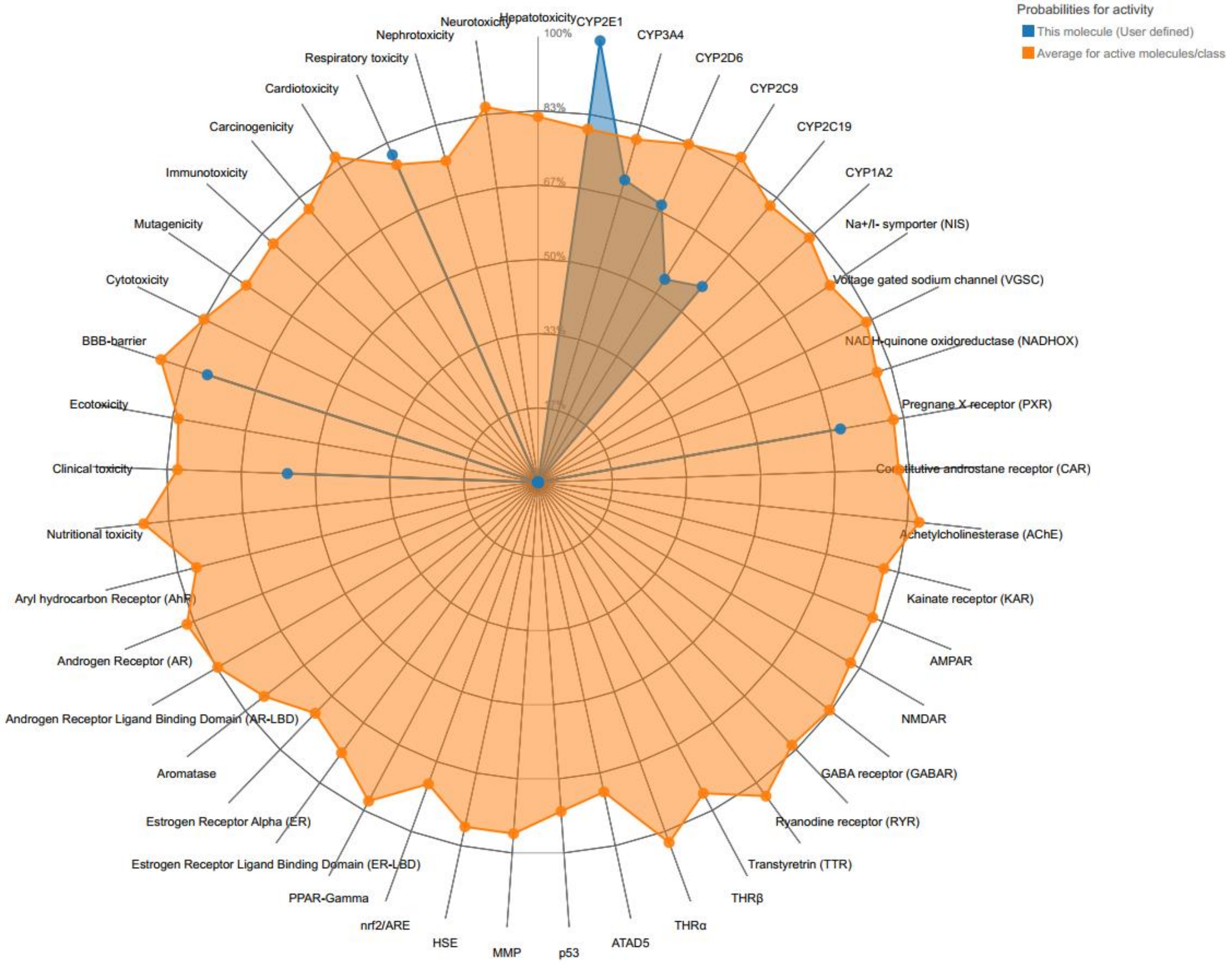

Figure S1a: Toxicity profile of Compound **19.**

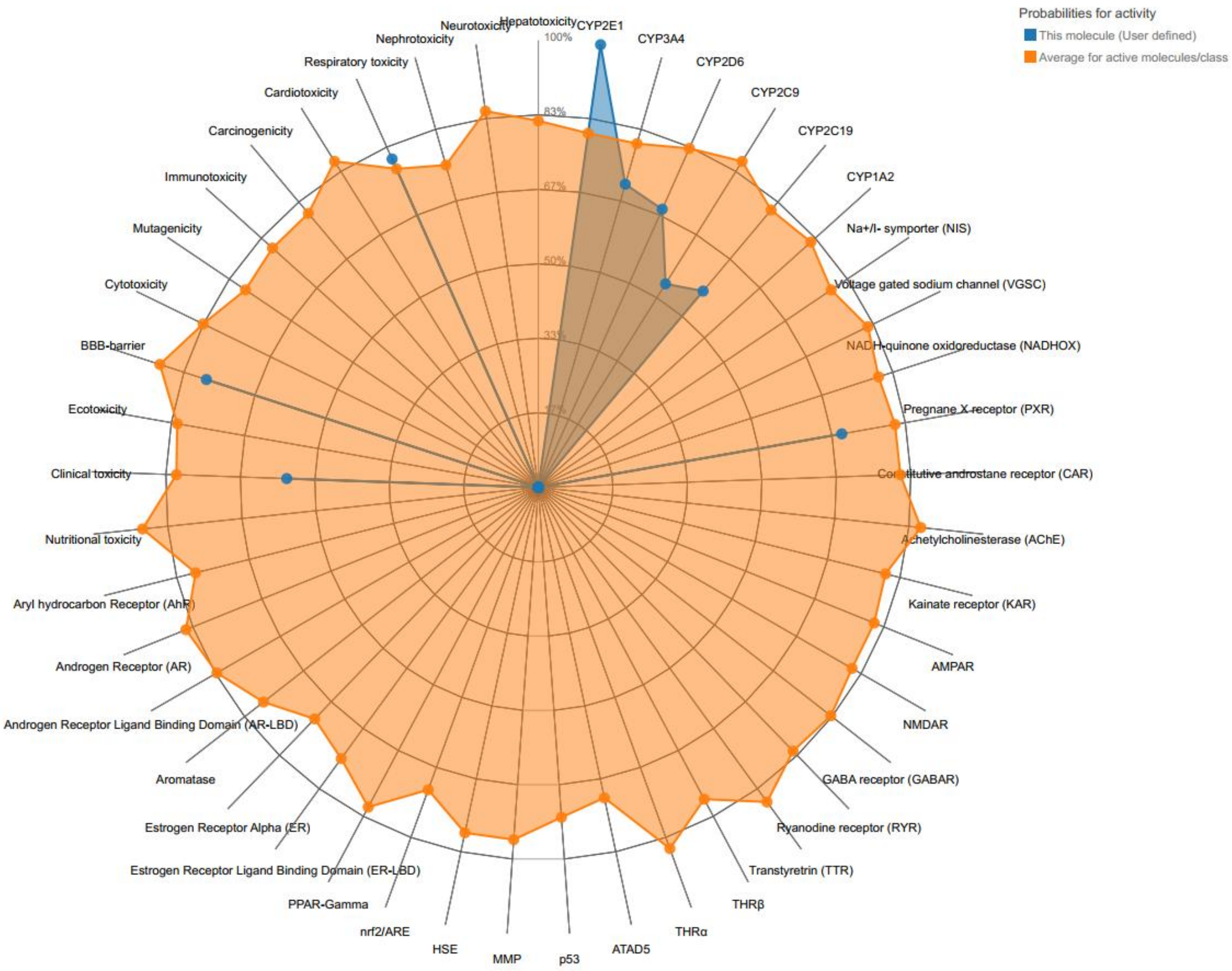

Figure S1b: Toxicity profile of Compound **20.**

Table S4: ADMET profiles of the designed compounds.

Comp	QED	logS	Synth	caco2	logD	BBB	logP	Neurotoxicity-DI	Ototoxicity	Hematotoxicity	Nephrotoxicity-DI	Genotoxicity
**9**	0.530	-5.270	2.345	-4.984	3.213	0.824	4.011	0.926	0.472	0.482	0.479	0.978
**10**	0.783	-3.522	3.883	-4.737	2.384	0.965	2.203	0.716	0.463	0.713	0.628	0.826
**11**	0.470	-5.705	2.073	-4.976	3.747	0.766	5.126	0.959	0.757	0.681	0.751	0.971
**12**	0.368	-5.318	2.365	-4.844	3.303	0.887	4.382	0.937	0.842	0.766	0.986	0.947
**13**	0.322	-5.468	2.385	-4.885	3.374	0.908	4.762	0.932	0.832	0.756	0.984	0.917
**14**	0.279	-5.614	2.407	-4.937	3.409	0.901	5.033	0.926	0.829	0.749	0.979	0.870
**15**	0.266	-7.508	2.499	-4.770	3.808	0.012	6.255	0.898	0.634	0.757	0.947	0.989
**16**	0.266	-5.122	2.665	-5.037	4.519	0.200	6.835	0.906	0.965	0.701	0.991	0.868
**17**	0.233	-6.612	2.876	-4.953	4.785	0.108	7.979	0.983	0.920	0.873	0.993	0.962
**18**	0.394	-5.011	2.635	-4.965	4.207	0.150	6.174	0.861	0.953	0.582	0.982	0.980
**19**	0.520	-4.698	2.268	-4.956	3.703	0.647	4.385	0.603	0.578	0.781	0.931	0.804
**20**	0.463	-4.898	2.292	-5.000	3.748	0.768	4.556	0.553	0.537	0.722	0.936	0.692
**21**	0.232	-4.764	2.375	-5.023	3.815	0.083	5.084	0.985	0.788	0.780	0.982	0.984
**22**	0.125	-5.847	2.761	-4.990	4.316	0.005	5.996	0.997	0.956	0.931	0.999	0.985
**23**	0.178	-5.330	2.487	-4.998	3.970	0.843	5.316	0.988	0.729	0.629	0.965	0.971
**24**	0.142	-6.226	2.578	-4.958	4.442	0.912	6.621	0.988	0.723	0.585	0.963	0.992

S4. Molecular Docking Details

Table S5: Docking validity evaluation

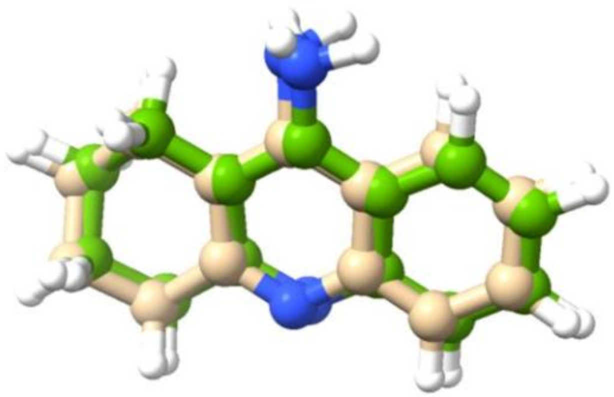

Ligand:Protein	Tacrine:AChE
RMSD	0.362
Legend	**Green:** Ligand obtained from Crystal<br>**Brown:** Docked ligand

Table S6: Docking results of designed compounds anti-Alzheimer activity.

**Ligands**	**Binding Affinity (ΔG) (kcal/mol)**
**13**	-11.1
**14**	-10.6
Tacrine	-9.0
